# Screen of Non-annotated Small Secreted Proteins of *Pseudomonas syringae* Reveals a Virulence Factor That Inhibits Tomato Immune Proteases

**DOI:** 10.1371/journal.ppat.1005874

**Published:** 2016-09-07

**Authors:** Takayuki Shindo, Farnusch Kaschani, Fan Yang, Judit Kovács, Fang Tian, Jiorgos Kourelis, Tram Ngoc Hong, Tom Colby, Mohammed Shabab, Rohini Chawla, Selva Kumari, Muhammad Ilyas, Anja C. Hörger, James R. Alfano, Renier A. L. van der Hoorn

**Affiliations:** 1 Plant Chemetics lab, Max Planck Institute for Plant Breeding Research, Cologne, Germany; 2 Center for Plant Science Innovation and the Department of Plant Pathology, University of Nebraska-Lincoln, Lincoln, Nebraska, United States of America; 3 Department of Plant Biology, University of Szeged, Szeged, Hungary; 4 Plant Chemetics lab, Department of Plant Sciences, University of Oxford, Oxford, United Kingdom; 5 Mass Spectrometry Group, Max Planck Institute for Plant Breeding Research, Cologne, Germany; University of Toronto, CANADA

## Abstract

*Pseudomonas syringae* pv. *tomato* DC3000 (PtoDC3000) is an extracellular model plant pathogen, yet its potential to produce secreted effectors that manipulate the apoplast has been under investigated. Here we identified 131 candidate small, secreted, non-annotated proteins from the PtoDC3000 genome, most of which are common to Pseudomonas species and potentially expressed during apoplastic colonization. We produced 43 of these proteins through a custom-made gateway-compatible expression system for extracellular bacterial proteins, and screened them for their ability to inhibit the secreted immune protease C14 of tomato using competitive activity-based protein profiling. This screen revealed C14-inhibiting protein-1 (Cip1), which contains motifs of the chagasin-like protease inhibitors. *Cip1* mutants are less virulent on tomato, demonstrating the importance of this effector in apoplastic immunity. Cip1 also inhibits immune protease Pip1, which is known to suppress PtoDC3000 infection, but has a lower affinity for its close homolog Rcr3, explaining why this protein is not recognized in tomato plants carrying the *Cf-2* resistance gene, which uses Rcr3 as a co-receptor to detect pathogen-derived protease inhibitors. Thus, this approach uncovered a protease inhibitor of *P*. *syringae*, indicating that also *P*. *syringae* secretes effectors that selectively target apoplastic host proteases of tomato, similar to tomato pathogenic fungi, oomycetes and nematodes.

## Author Summary

The extracellular space in the leaf (the apoplast) is colonized by a diversity of microbes that will have to deal with host-secreted hydrolytic enzymes, many of which accumulate during defence responses. We hypothesize that in addition to fungal and oomycete pathogens, the bacterial model plant pathogen *Pseudomonas syringae* also protects itself in the apoplast by secreting inhibitors targeting these apoplastic hydrolases. The genome of *P*. *syringe* harbours over 131 genes encoding putative small, non-annotated secreted proteins that have not been characterized previously. Here, we produced and purified 43 of these small proteins and tested them for their ability to inhibit the secreted immune protease C14 of tomato. We discovered a C14 protease inhibitor, coined Cip1, which carries chagasin-like motifs and contributes to virulence. Cip1 also effectively inhibits Pip1, another immune protease of tomato, known to suppress *P*. *syringae* infection. Interestingly, Cip1 has a lower affinity for the immune protease Rcr3, explaining why this protein, and PtoDC3000 producing Cip1, is not recognized in tomato plants carrying the *Cf-2* resistance gene, which uses Rcr3 as a co-receptor to detect pathogen invasion.

## Introduction

*Pseudomonas syringae* is an important model system for plant-pathogen interactions. Different pathovars of this Gram-negative bacterium can cause disease on a broad variety of plants. Most intensively studied is pathovar *tomato* DC3000 (PtoDC3000), which causes bacterial speck disease on tomato and Arabidopsis [1, 2]. This pathogen can enter the extracellular space (apoplast) of leaves through stomata and colonizes the apoplast, causing black specks, hence the name bacterial speck disease [1, 2]. *P*. *syringae* manipulates its host using effectors, which are secreted metabolites or proteins that manipulate the host cell. Most intensively studied are the type-III (T3) effectors that are injected into host cells through the T3 secretion system (T3SS) [3, 4]. These T3 effectors are collectively required but individually not essential to cause disease [5].

Filamentous tomato pathogens secrete dozens of apoplastic effectors with different functions, often contributing to pathogen virulence. The fungal tomato pathogen *Cladosporium fulvum*, for example, secretes Avr4 to prevent degradation of chitin in the fungal cell walls by secreted host chitinases [6]. *C*. *fulvum* also secretes Ecp6 to sequester chitin fragments and prevent their detection [7], and Avr2 to inhibit secreted host proteases [8]. Likewise, the oomycete tomato pathogen *Phytophthora infestans* secretes Epi and EpiC proteins inhibiting secreted host serine and cysteine proteases, respectively [9, 10]. In other pathosystems, apoplastic effectors include *P*. *sojae* Gip1, which inhibits a secreted glycosidase of its host, soybean [11] and *Ustilago maydis* Pep1, which blocks the apoplastic peroxidase of its host, maize [12]. Hence, secreted effectors are commonly used to manipulate the host apoplast. Importantly, all of these apoplastic effector proteins are small and often share no or low homology with annotated proteins.

The production of apoplastic effectors by filamentous pathogens suggests that also bacterial pathogens may employ apoplastic effectors to inhibit harmful enzymes in the apoplast. Here, we mined the genome of the model pathogen PtoDC3000 for genes encoding potential apoplastic effectors and found that many of these putative effectors are common to Pseudomonas species and expressed during apoplast colonization. We expressed over 40 of these non-annotated putative effectors as soluble proteins and screened them using competitive activity-based protein profiling (ABPP, [13]) for the inhibition of the C14 immune protease of tomato. Our results revealed that one of these proteins can inhibit immune proteases of tomato and contributes to virulence. This study investigates a repertoire of new putative effector proteins and describes the targets of the first apoplastic effector for this important model plant pathogen.

## Results

### *In silico* selection of non-annotated small secreted putative proteins

**Fig 1:**
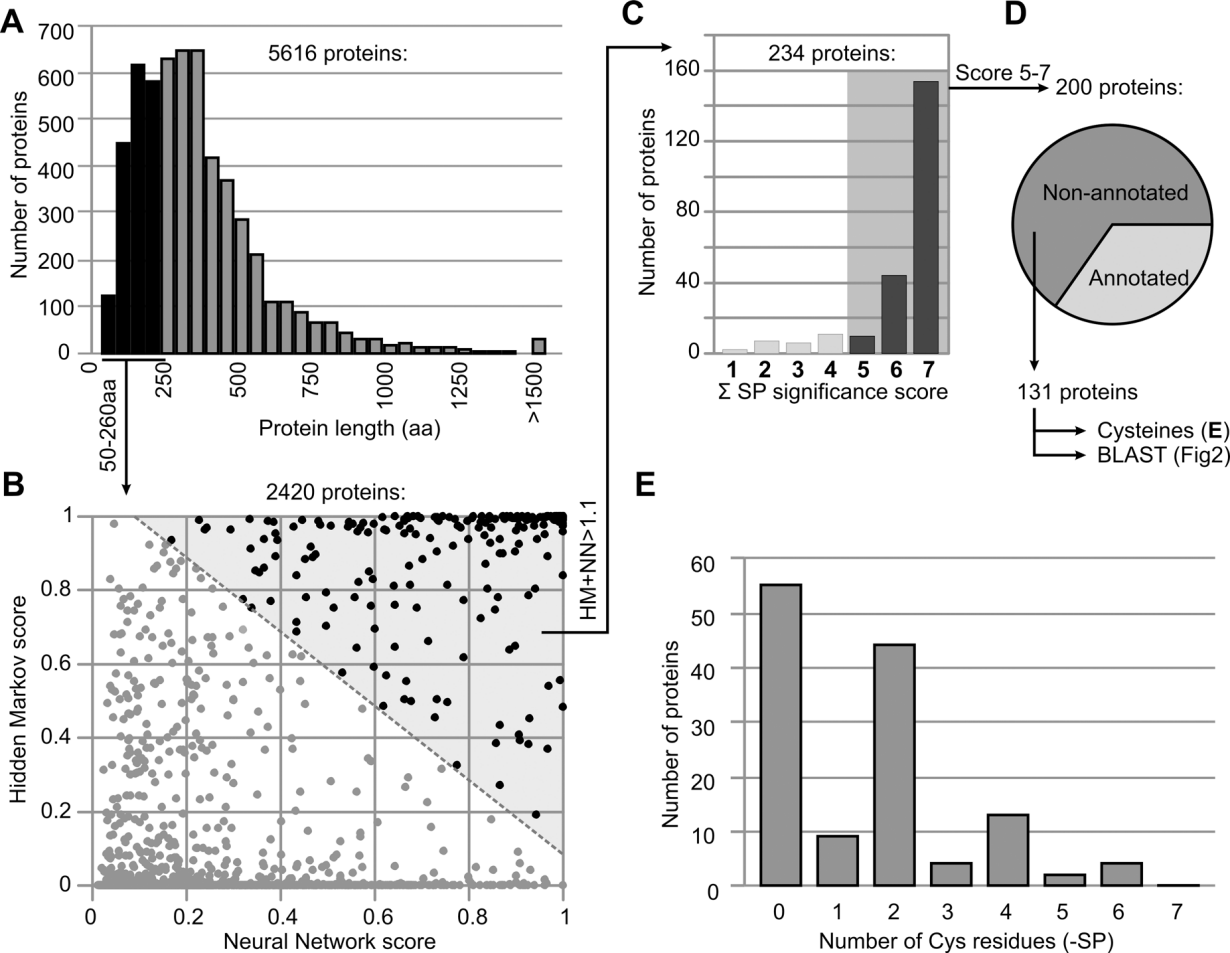
*In silico* selection of small, secreted, non-annotated proteins of PtoDC3000. (A) Selection of small proteins. 5616 proteins encoded by the PtoDC3000 genome were ranked based on amino acid length. 2420 proteins with 50–260 amino acid length were selected. (B) Selection of secreted proteins. 2420 small proteins were plotted with their SignalP scores for both Hidden Markov (HM) and Neural Network (NN). 243 proteins with HM+NN>1.1 were selected. (C) Selection of significant signal peptide (SP) prediction scores. For each of the 243 proteins, significance scores for both HM and NN algorithms were added up and 200 proteins with scores of 5 or higher were selected. (D) Selection of non-annotated proteins. Annotations in the genome database were used to select 131 non-annotated proteins. (E) Number of Cys residues in the selected 131 mature proteins. The predicted signal peptide was removed from the sequence and the number of Cys residues in the remaining protein was counted and plotted in a frequency histogram.

To identify non-annotated small secreted proteins of PtoDC3000, we analyzed the 5616 predicted proteins encoded by the PtoDC3000 genome ([14], **S1 Table**). First, we ranked the 5616 proteins on the length of the proteins, resulting in a histogram that visualizes that the majority of the PtoDC3000 proteins are 150–400 aa in length (**Fig 1A**). From this list we selected 2420 proteins with a length of 50–260 amino acids, which corresponds to protein sizes of 5–25 kDa. Most of the published apoplastic effectors fall in this size region.

Second, we analyzed these 2420 proteins for the presence of a Gram-negative bacterial signal peptide using SignalP [15]. SignalP predicts signal peptides using two algorithms: Hidden Markov (HM) and Neural Network (NN). Because the HM and NN algorithms predict signal peptides independently, we plotted each of the 2420 proteins against their scores in a dot plot (**Fig 1B**). To ensure that we select proteins that are likely to have a functional signal peptide, we selected the proteins that have an additive score of HM+NN>1.1. A total of 234 proteins were selected this way (**Fig 1B**). Third, the HM and NN algorithms also produce a significance score (HM (0–2) and NN (0–5)), which we used for further selection. By selecting proteins with a sum of both significance scores being 5 or higher, we selected 200 proteins having the highest confidence for secretion (**Fig 1C**).

Fourth, we investigated the 200 selected proteins for their annotation in the Pseudomonas genome database. Of the 200 small putative proteins, 69 are annotated, e.g. as components of secretion or motility systems (**S1 Table**). This group also includes chaperones, prolyl isomerases, various transporters, and a superoxide dismutase, carbonic anhydrase, and sorbitol dehydrogenase. None of these proteins are annotated as hydrolase inhibitor. The 131 remaining small proteins have previously only been annotated as ‘hypothetical proteins’ or ‘lipoproteins’, which means that they carry a lipobox after the signal peptide (**Fig 1D**).

To investigate if these 131 non-annotated proteins can be genuine proteins, we counted the number of cysteine residues in each mature protein, after omitting the signal peptide. The rationale being that secreted proteins frequently have disulphide bridges to increase their stability, and secreted effectors should therefore possess an even number of cysteine residues. Our analysis revealed that 47% of the 131 mature proteins have indeed an even number of cysteines, whereas only 11% have an odd number of cysteines (**Fig 1E**). The other 42% do not contain cysteines in the putative mature protein domain. These data suggest that our 131 non-annotated proteins represent a genuine set of secreted proteins and that a large portion of these putative proteins is equipped with putative disulphide bridges to provide stability in the apoplast.

**Fig 2:**
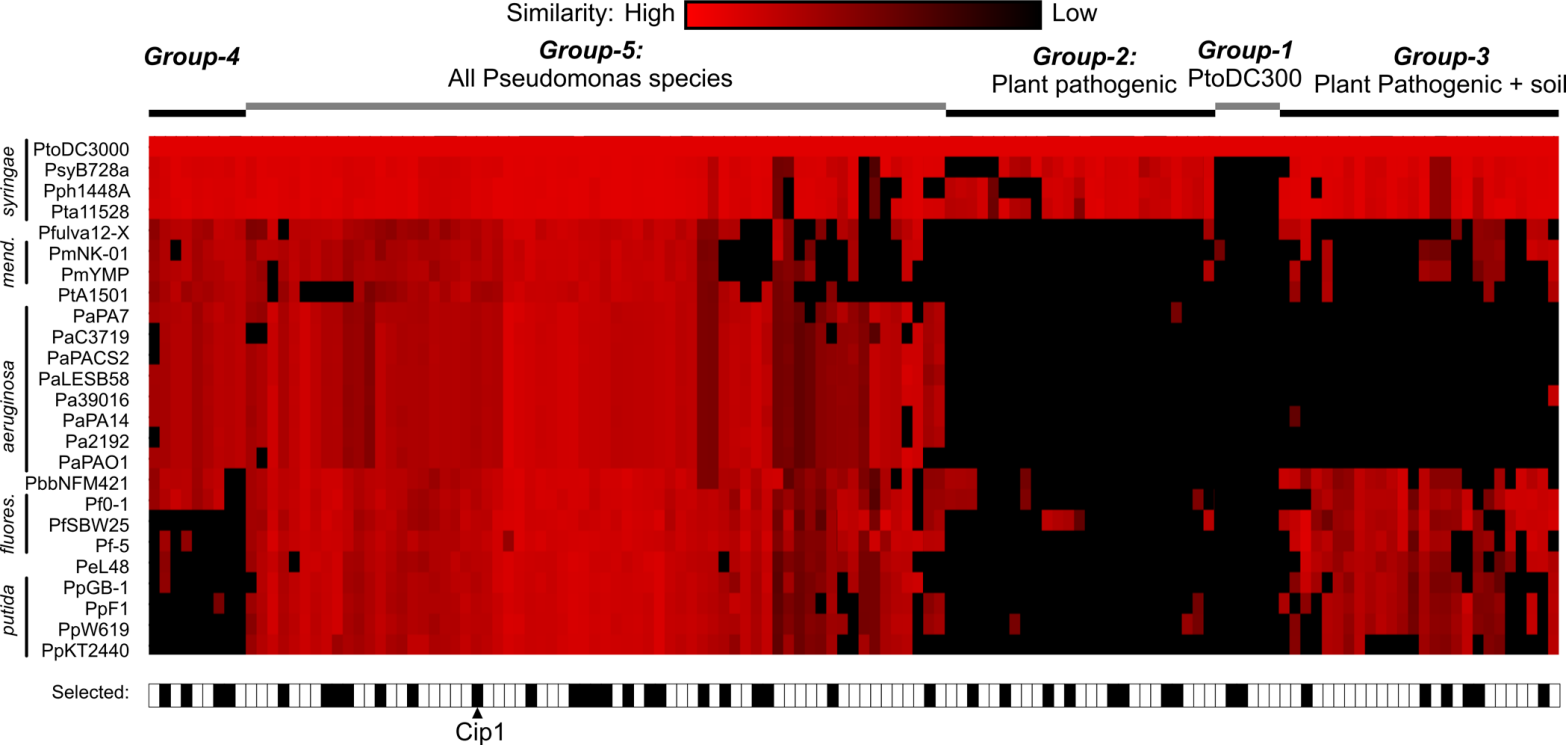
Occurence of small, secreted non-annotated proteins of PtoDC3000 in other *Pseudomonas* species. BLAST scores were generated for each of the 131 small secreted, non-annotated proteins against each of the protein databases of 24 sequenced *Pseudomonas* species. BLAST scores were presented in shades of red, and black boxes represent no significant BLAST score. Blast scores are clustered over both the species and proteins. Conservation of the proteins occurs in five groups (1–5). Representative putative proteins having the highest SP scores were picked from each of these classes and produced and purified (black boxes on the bottom). See **S1 Fig** for more details.

Next, we investigated how common these putative proteins are amongst Pseudomonas species. We selected 24 other Pseudomonas strains of which genome sequences are publicly available. This collection included three other plant pathogenic *P*. *syringae* strains: pathovars *syringae* B728a (PsyB728a), *phaseolicola* 1448A (Pph1448A) and *tabaci* 11528 (Pta11528), which are pathogenic on snap bean, soybean, and tobacco, respectively [14, 16–17]. The genome collection also included genomes of the rice and rapeseed epiphytes (*P*. *fulva* and *P*. *brassicacea*, respectively), twelve human epiphytes and opportunistic pathogens (*P*. *mendocina*, *P*. *stutzeri*, and *P*. *aeruginosa*) and eight soil bacteria (*P*. *fluorescence*, *P*. *entomophila* and *P*. *putida*). BLAST searches of the 131 selected putative secreted small non-annotated proteins against these 24 Pseudomonas genomes revealed that most putative proteins have clear homologs in other Pseudomonas species (**Fig 2** and **S1 Fig**). Interestingly, these homologies cluster in five groups. Group-1 consists of six putative proteins that are unique to PtoDC3000. Group-2 consists of 25 putative proteins that have close homologs only in other plant pathogenic bacteria. Group-3 consists of 26 putative proteins common with plant pathogenic bacteria and soil bacteria. Group-4 consists of nine putative proteins shared with opportunistic human pathogens, whilst the largest group (Group-5) consists of 65 putative proteins that are common to all Pseudomonas species. The high degree of conservation amongst Pseudomonas species suggests that these 131 putative proteins are genuine, *bona fide* proteins and are not incidentally generated by an occasional misannotation in the PtoDC3000 database. Of these, we randomly selected 43 putative proteins having relatively high SP confidence scores and we produced and purified these putative proteins for further studies (bottom of **Fig 2**).

### Several selected proteins are produced during apoplast colonization

**Fig 3:**
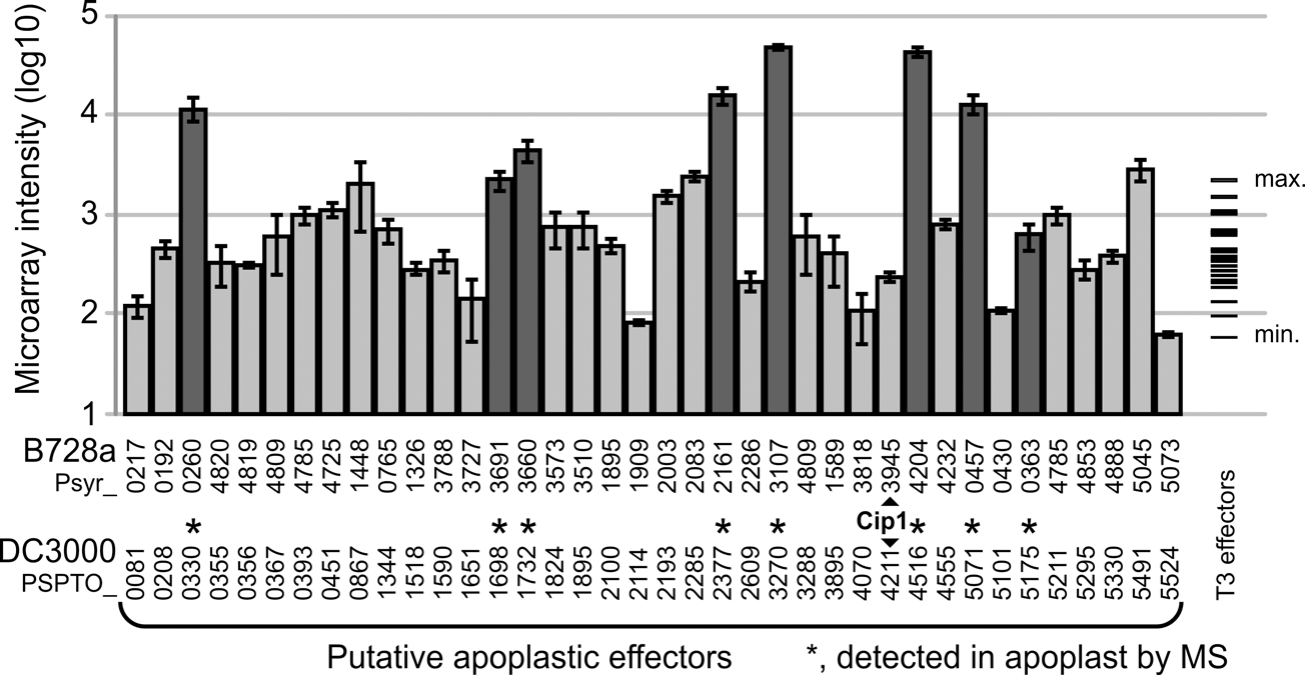
Expression and presence of putative effectors in the apoplast. The closest PsyB728a-homologs of the 43 small secreted, nonannotated proteins of PtoDC3000 proteins (bottom), were identified in the PsyB728a genome. The respective expression levels of these genes during colonization of PsyB728a in the apoplast were extracted from the microarray database. For comparison, also the expression levels of 25 type-III (T3) effectors of PsyB728a are shown on the right. Error bars indicate standard error of n = 4 biological replicates. PtoDC3000 proteins that have been detected in the apoplast by mass spectrometry are marked with an asterisk (dark grey bars).

To investigate if the genes encoding the selected 43 proteins are also expressed during infection, we mined gene expression databases for *P*. *syringae* infections. Infection with PsyB728a has been investigated for bacterial gene expression during epiphytic and apoplastic colonization of bean [18]. For the 38 of the 43 putative selected small non-annotated proteins, the ortholog was identified in the PsyB728a genome (**Fig 3**). For relative comparison, we also extracted the expression levels of the 25 type-III effectors of PsyB728a [19] from the same gene expression database [18]. The majority (21/38) of the selected genes encoding putative small, secreted non-annotated proteins are expressed with higher transcript levels than the average transcript levels of type-III effectors (**Fig 3**). It is therefore likely that many of the 43 selected genes are expressed during infection.

To also investigate if these proteins accumulate in the apoplast, we performed proteomic analysis of apoplastic fluids extracted from PtoDC3000-infected plants. This approach is challenging because small proteins may not produce two or more unique peptides for robust identification. Nevertheless, we could detect eight of the 43 selected proteins (dark gray bars and asterisks in **Fig 3**). Interestingly, these eight correspond to the genes with the highest transcript levels, often being higher than the highest expression level of type-III effectors genes, indicating that gene expression levels of the remaining genes are probably too low to detect the gene products by proteomics. In conclusion, the transcript levels and detection by proteomics indicates that several selected proteins are expressed by PtoDC3000 during apoplast colonization, and present in the apoplast.

### Efficient protein production using an adjusted bacterial expression system for secreted proteins

**Fig 4:**
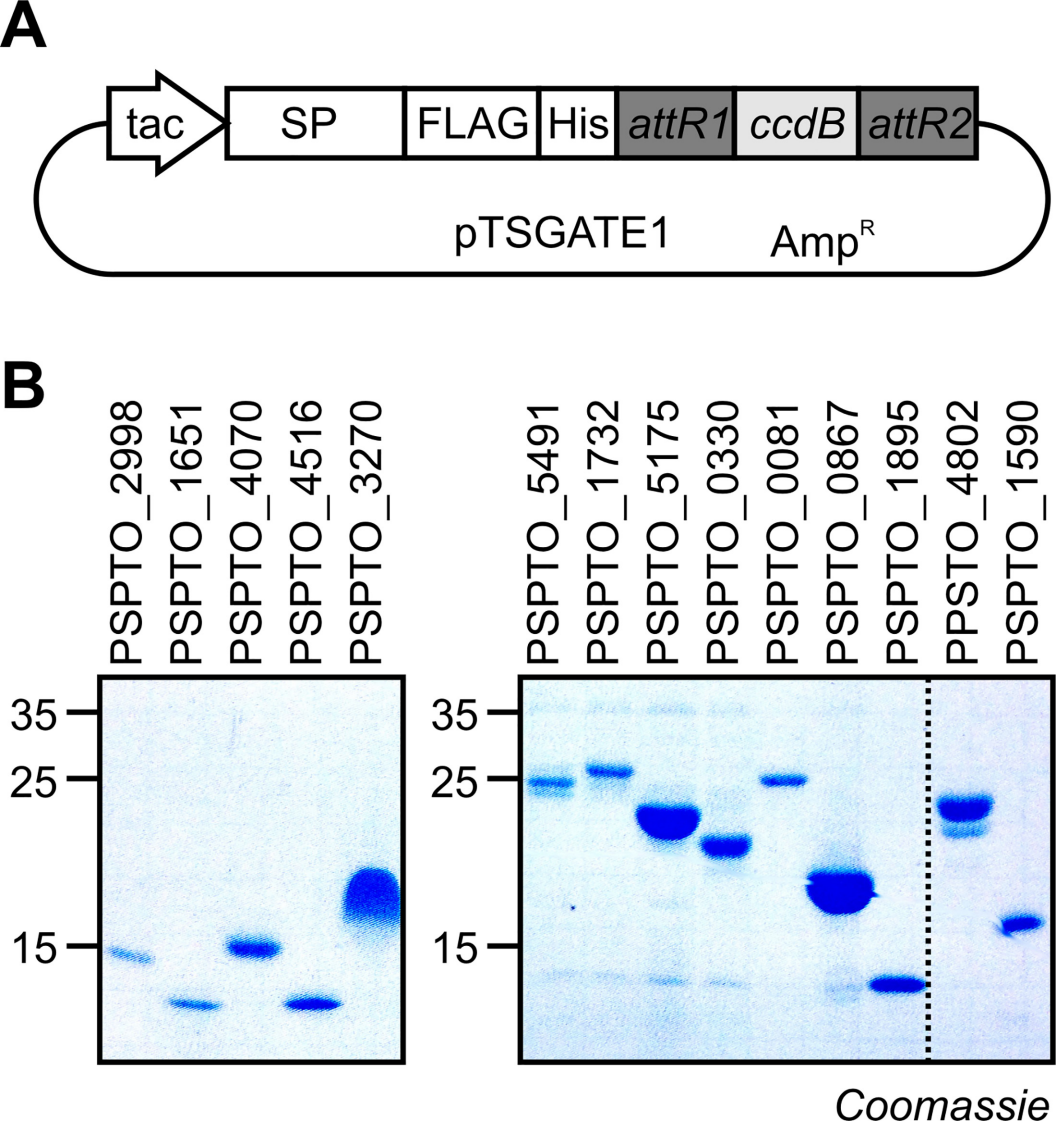
Heterologous production of secreted proteins using a Gateway-modified vector. (A) Gateway compatible vector pTSGATE1 for the expression of secreted FLAG/His-tagged proteins in *Escherichia coli*. The vector carries a Gateway cloning cassette with the *ccdB* suicide gene and the *attR1* and *attR2* recombination sites, preceded by a sequence encoding a secretion peptide (SP), a FLAG epitope tag for detection, and a Histidine-tag for purification. (B) Examples of secreted proteins purified from the medium of *E*.*coli* cells carrying pTSGATE1 expressing genes of interest. *E*. *coli* cultures were induced with IPTG, cleared by centrifugation and His-tagged proteins were purified from the medium by Nickel affinity chromatography.

To produce the selected proteins heterologously, we took advantage of the commercially available pFLAG-ATS expression system in *Escherichia coli* (Sigma-Aldrich). This expression system secretes N-terminally FLAG-tagged proteins into the growth medium using an N-terminal OmpA signal peptide to facilitate secretion. The growth medium of *E*. *coli* cultures has relatively low protein content and is easy to collect as supernatant after centrifugation, making this expression system ideal to produce small secreted bacterial proteins. Because of the large number of proteins, we decided to use the Gateway cloning strategy and add an N-terminal His tag to simplify the purification. We therefore generated a derivative of pFLAG-ATS that carries an extra fragment encoding a His-tag and the *ccdB* suicide gene located between the two *attR1* and *attR2* recombination sites (pTSGATE1, **Fig 4A** and **S1 File**). This construct was used for the expression and purification of 43 soluble proteins in a single step to a scale of over 100 μg per protein. Detection of the purified proteins on coomassie gels and anti-FLAG western blots confirmed the purity and molecular weight of these proteins (**Fig 4B** and **S2 Fig**).

### Screen for inhibitors of host immune protease identifies novel C14 inhibitor

Various tomato pathogens secrete apoplastic effectors that target papain-like cysteine proteases (PLCPs) that are secreted by the host during the immune response [8, 10, 20]. We therefore hypothesized that also *P*. *syringae* might employ this strategy and secrete protease inhibitors during infection. We first tested if any of the putative effectors would inhibit the secreted immune protease C14 of tomato. C14 is targeted by both EpiC1 and EpiC2B proteins of *P*. *infestans* [21] and is inhibited by Avr2 of *C*. *fulvum* [22]. C14 is also targeted by the RxLR-type effector AvrBlb2 of *P*. *infestans*, which blocks C14 secretion [23]. Furthermore, C14 knock-down dramatically increases *P*. *infestans* susceptibility [21], whilst C14 overexpression increases resistance [23]. Because C14 is a ‘hub’ for multiple effectors and because it plays a role in immunity, we decided to test if any of the 43 purified putative apoplastic effectors of PtoDC3000 would inhibit C14.

We screened the 43 proteins for their ability to block the activity of the mature C14 protease using competitive ABPP. Competitive ABPP is based on a preincubation of a protease with a putative inhibitor, followed by labeling of the non-inhibited proteases with an activity-based probe that reacts with the active site of the enzyme. Competitive ABPP has been routinely used to uncover the targets of Avr2, Epic1, Epic2B, Gr-Vap-1, CC9, and Pit2 [8, 10, 18–20, 24–29]. To facilitate quantification and medium-throughput screening, we used MV201, a fluorescent probe based on the papain inhibitor E-64 [30].

**Fig 5:**
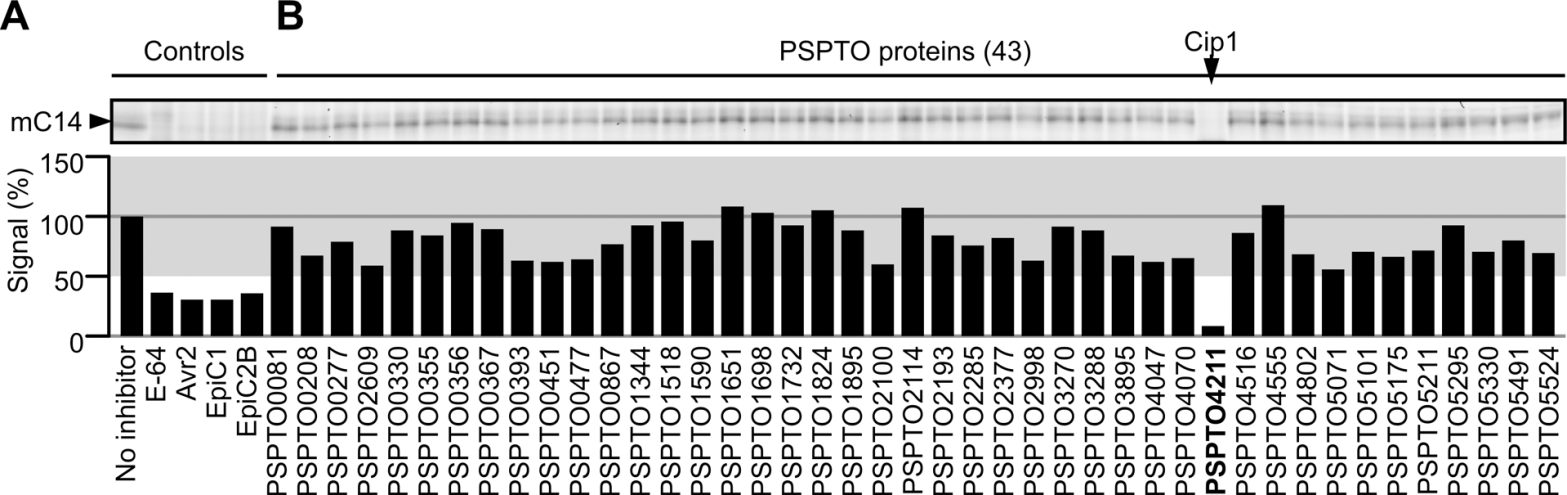
Competitive ABPP screen reveals inhibitor of immune protease C14. Protein extracts of leaves transiently overexpressing the C14 immune protease were pre-incubated with or without 50 μM E-64 or with 1 μg purified Avr2, EpiC1 or Epic2B (A) or 1 μg purified putative effector protein (B) or for 30 minutes and then incubated for one hour with 1 μM MV201 to label the non-inhibited proteases. Proteins were separated on a 12% protein gel and scanned for fluorescently labeled proteins. The signals were quantified and plotted in a bar graph. The region corresponding to 0.5 to 1.5 times the no-inhibitor-control is shaded grey. This screen was repeated twice with a similar outcome.

The C14 protease was transiently expressed by agroinfiltration of *Nicotiana benthamiana* [24]. Leaf extracts were labeled with MV201, separated on protein gels and fluorescently labeled C14 was detected by in-gel fluorescence scanning as a strong 30 kDa signal, representing the soluble, mature isoform of C14 (mC14 [21, 24]). This signal is absent in agroinfiltrated tissues that do not express C14 and in extracts pre-incubated with an excess of E-64 (**S3 Fig**). C14-containing extracts were diluted such that a robust fluorescent mC14 signal could still be detected upon MV201 labeling. Preincubation of the diluted mC14 with 1μg (50 μM) EpiC1 and EpiC2B blocked subsequent labeling by MV201 (**Fig 5A**), showing that mC14 inhibition by EpiCs can be detected using this competitive ABPP approach. To demonstrate that we could also detect interactions with weak inhibitors, we preincubated mC14 with 1 μg (33 μM) Avr2, a weak inhibitor of mC14 [21–22]. Importantly, Avr2 also prevents labeling of mC14 under these conditions (**Fig 5A**), indicating that we use conditions that allow us to detect even weak inhibitors of mC14.

We next screened the 43 proteins by preincubating 1μg (20–85 μM) of each protein with the mC14-containing leaf extract, followed by MV201 labeling. To select C14 inhibitors, signals were quantified and plotted for each PSPTO protein (**Fig 5B**). To exclude false positives, signals that were more than 0.5 times the signal of the no-inhibitor control were considered non-significant. Interestingly, this screen revealed one PtoDC3000 protein, PSPTO4211, that blocked labeling of mC14 by MV201 (**Fig 5B**). Hence, we named this protein C14-inhibiting protein (Cip1).

### *Cip1* contributes to virulence

**Fig 6:**
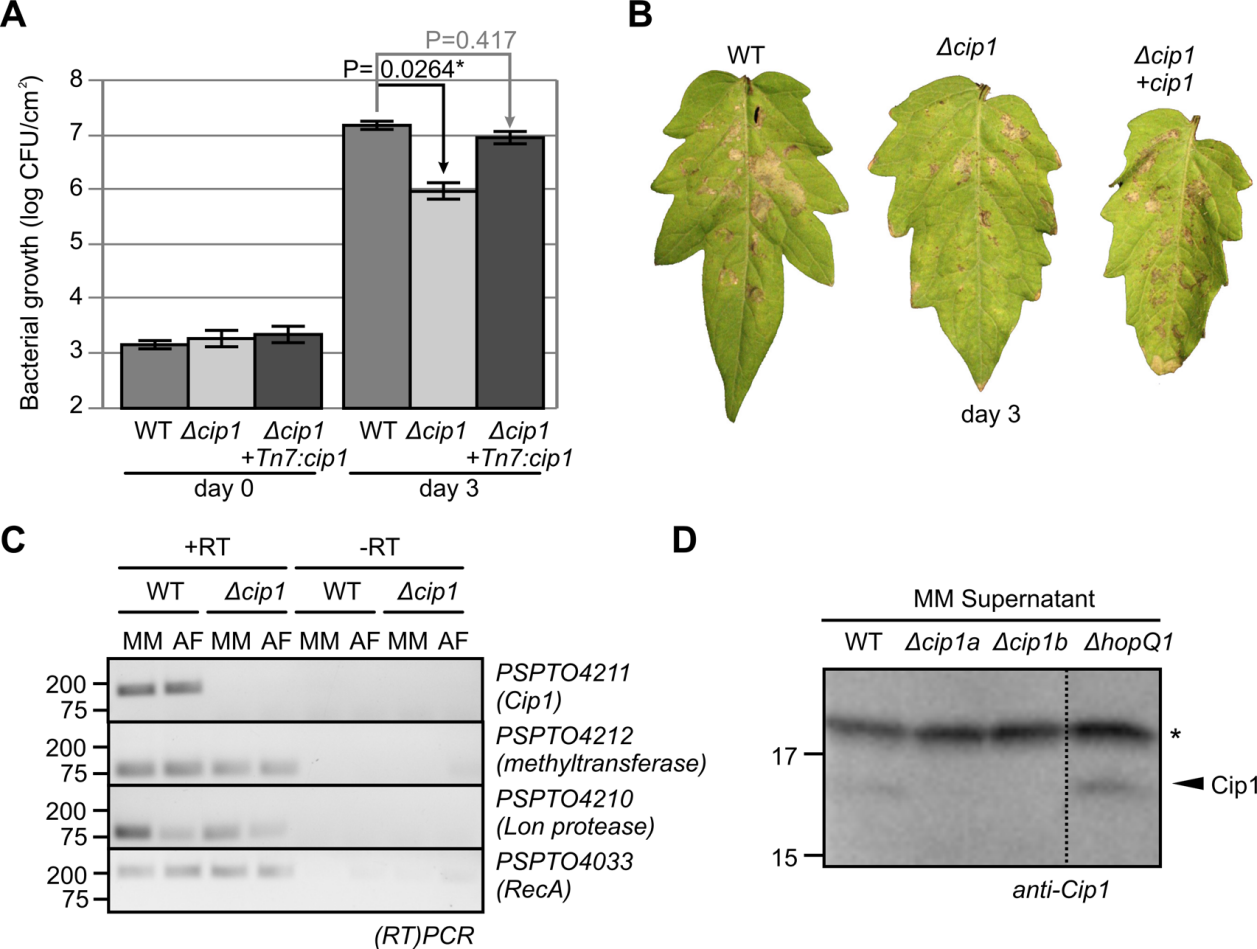
Cip1 is an extracellular virulence factor that is expressed during Infection. (A) The *Δcip1* mutant (UNL231) shows reduced bacterial growth in susceptible tomato plants when compared to wild type and *cip1*-complemented strains. Error bars represent standard errors of n = 4 biological replicates. A repetition experiment had a similar outcome. (B) The *Δcip1a* mutant (UNL231) has reduced bacterial speck symptoms when compared to wild type and complemented strains. Pictures were taken three days after bacterial infiltration with 10^5^ CFU/ml. (C) *Cip1* is expressed when bacteria are grown in minimal medium and *in planta*. RNA was isolated from PtoDC3000 and a *Δcip1* mutant (UNL232) grown in minimal medium (MM) containing mannitol-glutamate or isolated from apoplastic fluids (AF) at two days after infection, and used as template for PCR with and without reverse transcriptase (RT) pretreatment. Primer pairs were used to amplify *Cip1*, the two flanking genes and *RecA* as a control. (D) Cip1 protein is detected I the supernatant of cultures of bacteria grown in minimal medium. Wild-type, *Δcip1* (UNL231(a) and UNL232(b)) and *ΔhopQ1-1* mutant bacteria were grown in minimal medium (MM), centrifuged and the supernatant was used for western blot analysis using a specific antibody raised against the Cip1 protein produced in *E*. *coli*. *, background signal showing equal loading.

To determine the importance of *cip1* for the virulence of PtoDC3000, we generated two independent knock-out mutants of PtoDC3000 through homologous recombination (*Δcip1a* and *Δcip1b*) and generated complemented strains by transforming the *Δcip1* mutants with wild-type Cip1 using Tn7 transposition [31]. When infiltrated into tomato leaves, the *Δcip1* mutants grow significantly less when compared to wild-type PtoDC3000 (**Fig 6A** and **S4 Fig**). By contrast, both *Δcip1* mutant strains grow indistinguishable from wild type PtoDC3000 in liquid cultures (*in vitro*, **S5 Fig**). Importantly, the *in planta* growth defect is complemented in the *Δcip1* +Tn7:cip1 strain (**Fig 6A**). The differential bacterial growth of the strains correlates with the severity of the disease symptoms: *Δcip1* strains cause less bacterial spot symptoms than the wild-type or the complemented strains (**Fig 6B**). This experiment demonstrates that *cip1* encodes an important virulence factor for PtoDC3000 on tomato.

### *Cip1* is expressed during infection

The phenotype of the *Δcip1* mutant suggests that the *cip1* gene is expressed during infection. To confirm *cip1* expression, we performed semi quantitative RT-PCR on RNA extracted from wild-type and a *Δcip1* mutant PtoDC3000 grown in minimal medium (which mimics infection conditions) and isolated from the apoplast of infected plants, two days after infection. RT-PCR with *cip1*-specific primers amplified a gene product that is absent in the *Δcip1* mutant and if no reverse transcriptase was added (**Fig 6C**), demonstrating that *cip1* is expressed when bacteria are grown in minimal media or during infection. By contrast to *cip1* itself (*PSPTO4211*), the expression of *cip1*-flanking genes *PSPTO4210* and *PSPTO4212* is similar in the *Δcip1* mutant when compared to wild-type bacteria (**Fig 6C**), indicating that the flanking genes are unaffected in the *Δcip1* mutant.

### Cip1 protein can be detected extracellularly

Cip1 has a predicted signal peptide for secretion of 21 amino acids. To investigate if Cip1 protein can also be detected in the apoplast during infection, we raised a Cip1-specific antibody against the Cip1 protein and performed western blot analysis of apoplastic fluids isolated from tomato plants infected with wild-type and *Δcip1* mutant bacteria. Unfortunately, the affinity of the Cip1 antibody is not high enough to detect Cip1 in apoplastic fluids. We therefore also tested the supernatant of a centrifuged culture of wild-type and *Δcip1* mutant bacteria grown in minimal medium. Western blot analysis of these samples displayed a signal of the expected molecular weight in both the WT strain and the *ΔhopQ-1* mutant control that was absent in both tested *Δcip1* mutants (**Fig 6D**). These data demonstrate that Cip1 protein is detected in the medium of PtoDC3000 cultures and suggest that Cip1 occurs in the apoplast during infection.

We have performed several experiments to also investigate the suppression of apoplastic PLCPs during infection but failed to detect a consistent suppression using MV201 labeling on apoplastic fluids isolated from infected and non-infected plants (**S6 Fig**). We believe this is caused by the relatively low amount of Cip1 produced locally during infection when compared to the active PLCPs that are present in apoplastic fluids isolated from whole leaves.

### Cip1 is a Chagasin-like inhibitor of papain and C14

Analysis of the Cip1 protein sequence using PFAM revealed that this protein contains a chagasin motif, NPTTG. Chagasins are cysteine protease inhibitors initially described for the human protozoan parasite *Trypanosoma cruzi*, the causal agent of Chagas disease [32]. In *T*. *cruzi*, chagasin is an intracellular protein that controls the activity of cruzipain, an endogenous cysteine protease during the development of this parasite. Similar roles in regulation of endogenous proteases have been described for chagasin-like proteins from other human protozoan parasites [33–36].

**Fig 7:**
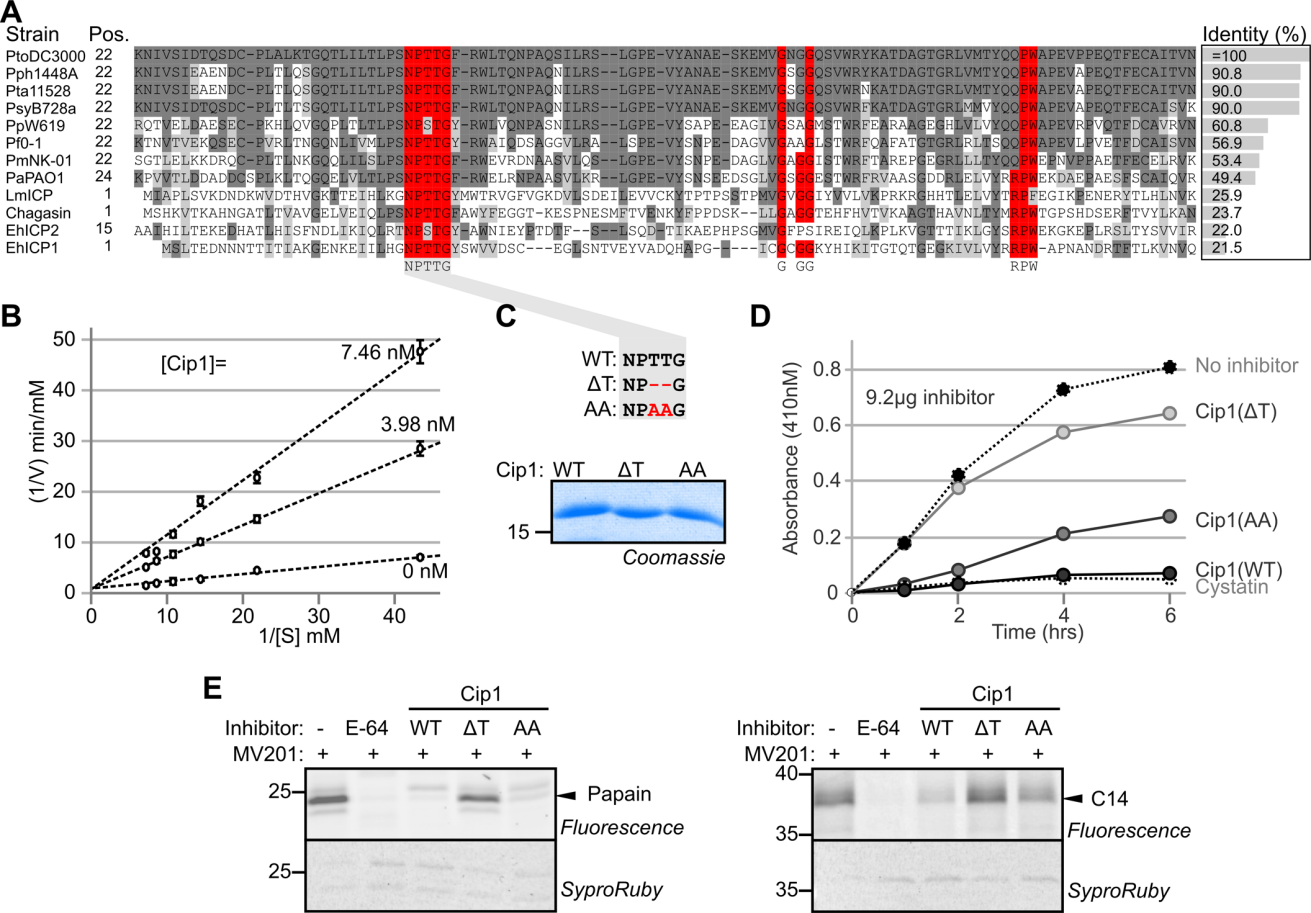
Cip1 is a Chagasin-like protease inhibitor. (A) Alignment of chagasins showing conserved chagasin motifs NPTTG, GxGG and RPW (red). Amino acid identity with Cip1 is shown next to each of the protein sequences. (B) Cip1 is a competitive inhibitor of papain. Papain was incubated without or with 7.46 or 3.98 nM Cip1 and assayed at increasing substrate concentrations. Fluorescence values were plotted against the inverse velocity (V) and inverse substrate concentration [S] in a Lineweaver- Burk plot. K_i_ = 3.98 nM. (C) Cip1 mutant proteins are soluble and stable. The NPTTG motif was mutated by removing the two threonines (ΔT) or replacing them by alanines (AA). Mutant and wild-type (WT) Cip1 proteins were expressed in *E*.*coli* as His-FLAG-tagged protein, purified, separated on SDS-PAGE and stained by coomassie. (D) Mutagenesis of the NPTTG motif affects inhibition of papain. 4.1 μM papain was incubated with 0.56 μM (mutant) Cip1 inhibitor or chicken cystatin and 0.2 mM BAPNA. Substrate conversion was monitored by increased fluorescence at 410 nm over time. (E) Protease activity profiling of papain (left) and C14 (right) in the presence of (mutant) Cip1 using pure papain or apoplastic fluids from leaves overexpressing C14 were preincubated with 100 μM E-64 or (mutant) Cip1 for 30 minutes, and then labeled with 1 μM MV201 for 1 hour.

Alignment of Cip1 protein sequence with homologs from other Pseudomonas species and chagasins of three human protozoan parasites shows that the homology of Cip1 is high to the homolog in other *P*. *syringae* strains (>90% identity) and moderately high (ca. 50% identity) to homologs from other Pseudomonas species, but Cip1 has less than 26% identity with the well-characterized chagasins from protozoans (**Fig 7A**). Nevertheless, in addition to the conserved NPTTG motif, two additional Chagasin motifs (GxGG and RPW) are conserved amongst these proteins. Importantly, the alignment also reveals that all Pseudomonas chagasins carry a putative signal peptide for secretion, whereas the protozoan chagasins do not (**Fig 7A**).

To confirm that Cip1 can inhibit papain-like proteases, we performed classical protease inhibition assays on purified papain using the chromogenic substrate BAPNA. Lineweaver-Burk plot analysis revealed that Cip1 is a classical, competitive inhibitor of papain with a K_i_ of 3.98 nM (**Fig 7B**).

To confirm that Cip1 is a chagasin-like protein, we mutated the conserved NPTTG motif by deleting the two conserved threonines (ΔT) or by substituting them into two alanines (AA). These mutations were previously shown not to affect the chagasin structure but significantly reduce the affinity of chagasin for papain [37]. The wild-type and mutant Cip1 proteins are soluble proteins and were purified using the N-terminal His-tag (**Fig 7C**). When compared to Cip1(WT), the Cip1(AA) substitution mutant has significantly less inhibitory activity, whereas the Cip1(ΔT) deletion mutant is even less able to inhibit papain (**Fig 7D**). This relative activity is consistent with the mutants described for chagasin [37]. To examine if this reduced activity is also displayed on C14, we preincubated apoplastic fluids of plants transiently overexpressing C14 with (mutant) Cip1 and then added MV201 to label the non-inhibited proteases. Papain was included as a control to compare with the traditional protease activity assay. Suppression of labeling shows that the Cip1(ΔT) deletion mutant has lost most of its inhibitory activity towards both papain and C14 whereas the Cip1(AA) substitution mutant is still able to partially suppress labeling of papain and C14 (**Fig 7E**). These data are consistent to the traditional substrate conversion assay (**Fig 7D**) and the previously described chagasin mutants [37]. In conclusion, the NPTTG chagasin motif is also important for Cip1 inhibitory activity to the same extend as chagasins, consistent with Cip1 being a chagasin-like protease inhibitor.

**Fig 8:**
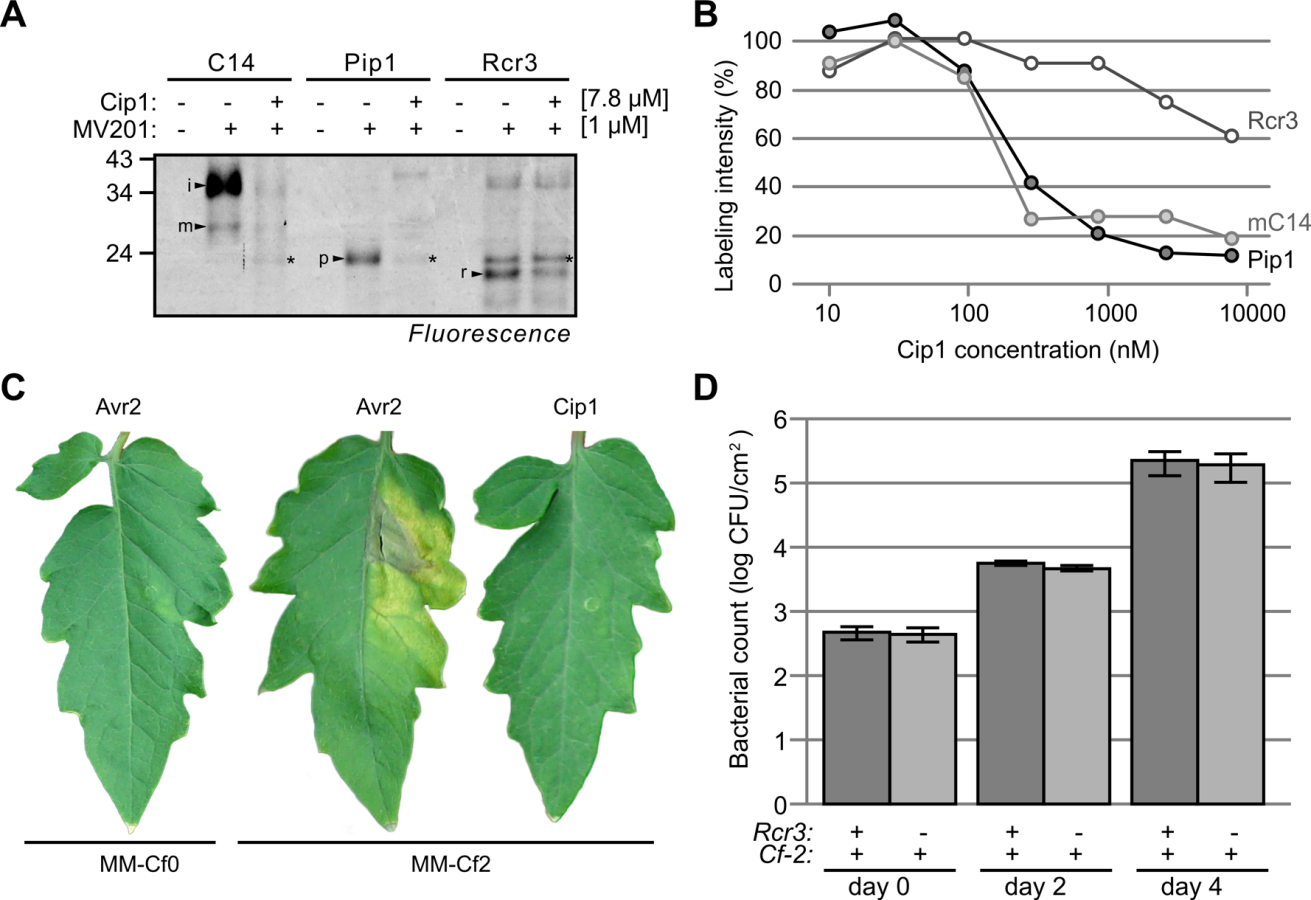
Cip1 blocks all three immune proteases of tomato with different affinities and escapes recognition by Rcr3/Cf-2. (A) Cip1 suppresses active site labeling of C14, Pip1 and Rcr3. Extracts containing the proteases were pre-incubated with 7.8 μM Cip1 and then labeled with MV201, a fluorescent probe for PLCPs. *, endogenously biotinylated protein. Abbreviations: i, iC14; m, mC14; p, Pip1; r, Rcr3. (B) Cip1 has highest affinity for Pip1 and C14. Extracts containing the proteases were pre-incubated with various concentration of Cip1 and then labeled with MV201. Labeled proteins were quantified from gel and plotted against the Cip1 concentration. (A-B) C14, Pip1 and Rcr3 were transiently overexpressed by agroinfiltration and proteomes were isolated and pre-incubated with and without various concentrations of Cip1 at pH 6.2 for 30 minutes and then labeled with 1 μM MV201 for one hour. Labeled proteins were detected by in-gel fluorescence scanning. (C) Cip1 does not trigger hypersensitive cell death in Cf2/Rcr3 tomato plants. 100 nM Avr2 or 1 μM Cip1 were injected into leaflets of MM-Cf2 and MM-Cf0 tomato plants and photographs were taken five days after the injection. (D) The presence of Rcr3 does not affect bacterial growth of PtoDC3000. Cf2/Rcr3 and Cf2/rcr3-3 tomato plants were spray-inoculated with PtoDC3000 and the bacterial populations were determined at 0, 2 and 4 days-post-inoculation. Error pars represent SEM for n = 4 different leaf samples taken during the same assay. This experiment was repeated four times with similar results.

We next tested if, in addition to mature C14, also the intermediate isoform of C14 can be inhibited by Cip1. Intermediate C14 (iC14) migrates at 35 kDa and differs from the 30 kDa mature C14 (mC14) by carrying a C-terminal granulin-like domain [21]. Since iC14 tends to precipitate, the activity of this isoform can be monitored in extracts that have not been centrifuged. Preincubation of non-centrifuged extracts containing iC14 with Cip1 also blocks labeling of iC14 (**Fig 8A**), indicating that Cip1 inhibits both isoforms of C14.

### Cip1 escapes recognition by the Cf-2/Rcr3 perception system

Other tomato papain-like proteases, such as Rcr3 and Pip1 are often targets of the same set of pathogen-derived inhibitors [20, 21, 24]. We therefore tested if Cip1 also inhibits these proteases. Both Pip1 and Rcr3 were produced by agroinfiltration in *N*. *benthamiana* and isolated from agroinfiltrated plants in apoplastic fluids. Labeling of diluted apoplastic fluids with MV201 causes the characteristic 25 and 23 kDa signals for Pip1 and Rcr3, respectively (**Fig 8A**). Preincubation with 7.8 μM Cip1 blocks Pip1 labeling and suppresses Rcr3 labeling, respectively (**Fig 8A**), indicating that Cip1 also inhibits Pip1 and Rcr3. All our assays are performed at apoplastic pH (pH 5–5.5), which is important since some inhibitors (e.g. Avr2, [8]) only inhibit at acidic and not at neutral pH. By contrast, inhibition of mC14, Pip1 and Rcr3 by Cip1 occurs at both apoplastic and neutral pH (**S7 Fig**).

The consistently weaker suppression of Rcr3 labeling, however, suggests that Cip1 is a weak inhibitor of Rcr3 when compared to Pip1 and C14. To further investigate the relative strength of inhibition, apoplastic fluids of plants transiently overexpressing Rcr3, C14 and Pip1 were pre-incubated with a dilution series of Cip1 and then labeled with MV201. The fluorescence intensity was measured from protein gels and plotted against the Cip1 concentration. This inhibitor dilution experiment revealed that Cip1 is a strong inhibitor of both C14 and Pip1, and a weak inhibitor of Rcr3, requiring at least 200-fold more Cip1 to reach the same suppression level for Rcr3 when compared to C14 and Pip1 (**Fig 7B**). We also tested an additional 34 natural Rcr3 variants [26] but we were unable to identify a natural variant of Rcr3 with increased sensitivity for Cip1 inhibition (**S8 Fig**).

When leaves of tomato plants carrying *Rcr3* and the *Cf-2* resistance gene were injected with Avr2 and Gr-Vap-1, a hypersensitive cell death was triggered by their ability to interact with Rcr3 [20, 38]. To test if Cip1 is also recognized in Money Maker Cf2 (MM-Cf2) tomato plants, which carry *Rcr3* and *Cf-2*, we injected Cip1 at protein concentrations up to 1 μM. However, neither hypersensitive cell death nor chlorotic responses were observed, even after prolonged incubation times (**Fig 7C**). By contrast, 100 nM Avr2 is sufficient to trigger hypersensitive cell death in MM-Cf2 tomato plants, but not MM-Cf0 tomato plants, which lack the *Cf-2* resistance gene (**Fig 7C**). This indicates that Cip1 is not recognized by MM-Cf2 tomato plants.

We also tested if the presence of Rcr3 had an effect on bacterial growth of PtoDC3000. We therefore performed bacterial growth assays on MM-Cf2 plants carrying the wild-type *Rcr3* gene (MM-Cf-2/*Rcr3* plants) or the *rcr3-3* null mutant gene (MM-Cf-2/*rcr3*-*3* plants, [39]). These bacterial growth assays showed that PtoDC3000 grows equally well on MM-Cf2/*Rcr3* as well as MM-Cf2/*rcr3-3* tomato plants (**Fig 7D**), consistent with the absence of Cip1 recognition by the *Cf-2/Rcr3* system.

## Discussion

Here we describe a diverse set of small secreted putative proteins encoded by the *P*. *syringae* genome that could act as effectors that manipulate the apoplast. We developed and employed an efficient expression system to produce these proteins heterologously and we used competitive ABPP to discover a novel chagasin-like protein that inhibits the activity of apoplastic immune proteases of tomato and contributes to virulence of PtoDC3000.

By choosing stringent selection criteria to select putative small non-annotated secreted proteins, we are relatively confident that these proteins are genuine proteins. First, the vast majority of these proteins are also predicted from genomes of other Pseudomonas species. Second, the observed high ratio of paired versus unpaired cysteines is not a random distribution and suggests that these proteins carry disulphide bridges, which would stabilize them upon secretion. Third, most genes are probably expressed during apoplast colonization at a higher level than T3 effectors and eight proteins with the highest expression levels were detected in the apoplast by mass spectrometry. Fourth, we were able to produce a large number of these putative proteins as soluble proteins, indicating that they were properly folded also upon heterologous expression.

The ability to produce these secreted proteins opens several research avenues to elucidate their functions. Although several of these proteins can have enzymatic or structural functions, we suspect, based on the small size, that many of these proteins are enzyme inhibitors that manipulate secreted host enzymes, such as glycosidases, proteases, peroxidases and lipases. For example, Gip1 from *Phytophthora sojae*, Avr2 from *Cladosporium fulvum*, Epi1 from *Phytophthora infestans* and Pep1 from *Ustilago maydis*, are secreted, pathogen-derived inhibitors that target host endoglucanases, cysteine proteases, subtilases or peroxidases, respectively [8–9, 11–12]. Some of the apoplastic effectors might interact with the components of the host or pathogen cell wall or membranes; retrieve metabolites or ions; or sequester elicitors to prevent perception. For example, *C*. *fulvum* secretes Avr4 which binds to the fungal cell wall to protect it against chitinases, and *C*. *fulvum* Ecp6, which sequesters chitin fragments to prevent their perception by the plant [6–7]. In addition to testing for inhibitors of apoplastic hydrolases, our FLAG-His-tagged proteins can be used for pull-down assays to identify interacting molecules, and to produce pure protein for crystallographic studies to elucidate their structures.

We employed a new approach to screen putative apoplastic effector proteins for novel functions by using competitive ABPP. Competitive ABPP is a powerful method to identify inhibitors as this assay can be performed in medium through-put and without the need to purify the enzyme and/or know their substrates. Cravatt and co-workers used library-to-library competitive ABPP screens to identify selective inhibitors for dozens of mammalian serine hydrolases [40]. Here, we used competitive ABPP to identify natural inhibitors secreted by a pathogen. Competitive ABPP assays are relatively quick and robust, and the throughput will increase further using broad range probes that label multiple enzymes simultaneously. The introduction of new activity-based probes for other apoplastic enzymes can now be used to identify PtoDC3000 apoplastic proteins that inhibit subtilases, lipases, acyltransferases and glycosidases [41–42].

Our competitive ABPP screen revealed that PtoDC3000 secretes the chagasin-like Cip1 that can inhibit tomato immune proteases. Chagasins have been mostly described for protozoan parasites. Protozoan chagasins are produced without signal peptide and control the activity of endogenous papain-like cysteine proteases that play essential roles during infection [32–36]. In recent years, however, these protozoan chagasins were found to be released during later stages of the infection and inhibit extracellular proteases of the host [43]. A role for bacterial chagasins has not yet been elucidated [44]. An argument supporting the hypothesis that Pseudomonas chagasins have extracellular targets lies in the fact that chagasins so far exclusively target C1A proteases, but that, even though all Pseudomonas species carry a chagasin ortholog, most Pseudomonas genomes do not encode C1A proteases [43–45]. Like Cip1, the chagasin ortholog of other pathogenic Pseudomonas bacteria may also act by suppressing secreted host proteases. Likewise, chagasins produced by soil Pseudomonas may act in controlling extracellular proteases that might be produced by the microbiome. The role of chagasins in other Pseudomonas species remains to be investigated.

Importantly, Cip1 contributes to virulence on tomato because *cip1* mutants show significantly reduced bacterial growth on this host and this phenotype can be complemented by transformation with wild type Cip1. This is consistent with a role for secreted immune proteases being harmful to PtoDC3000 because transgenic tomato lines with reduced Pip1 protein and activity levels are significantly more susceptible for PtoDC3000 infection [46]. Thus, Cip1 provides protection against secreted host proteases that would otherwise suppress pathogen growth.

We found that Cip1 inhibits Rcr3 less efficiently when compared to Pip1 and C14. Pip1 and Rcr3 are close homologs and it is therefore remarkable that Cip1 is able to distinguish between these proteases. Previously described pathogen-derived inhibitors Avr2, Gr-Vap-1, Epic1 and Epic2B, do not show such a distinct discrimination between Rcr3 and Pip1, even though they showed distinct affinities for the less related C14 [20–25]. The ability to distinguish between Pip1 and Rcr3 could be a strategy of PtoDC3000 to prevent recognition by Rcr3 while suppressing the dominant proteolytic activities of Pip1 and C14 in the apoplast during infection [25]. This strategy is different when compared to that used by *Phytophthora infestans*, which uses ‘stealthy’ EpiC inhibitors, which do inhibit Rcr3, but evade recognition [25]. The molecular basis for the evolution of this evasive chagasin-like effector that can distinguish between Rcr3 and Pip1 is yet another exiting topic for future studies.

## Materials and Methods

### *In silico* selection of secreted effector candidates

The predicted protein sequences of the PtoDC3000 genome and their annotations [14] were obtained from http://www.pseudomonas-syringae.org/. SignalP3.0 (http://www.cbs.dtu.dk/services/SignalP-3.0/) was used for signal peptide prediction [15] and both the scores for Hidden Markov and Neural Network (sum >1.1), as well as their significance scores (sum >4), were used to select secreted proteins. The annotation in the protein database was used to remove annotated proteins. The number of cysteines in each of these candidate proteins was counted after the removal of predicted signal peptide.

### Generating the BLAST matrix

The list of 234 candidate effectors was blasted against a database of predicted proteins from 25 different *Pseudomonas* strains (144,359 sequences total) using Blastp 2.2.26 and an initial e-value cut-off of .001 (correlating for most candidates to roughly 35% similarity). The blast results were parsed and a matrix was generated in which each candidate protein was assigned a vector of 25 values reflecting the percentage similarity between the candidate and the best matching protein from each of the strains. When no hit was found with e < .001, the percentage similarity was set to -1. Both the rows (candidates) and columns (strains) were hierarchically clustered using Cluster 3.0 [47] using a Euclidean distance metric and centroid linkage and the resulting trees were visualized using java TreeView (http://jtreeview.sourceforge.net).

### Extraction of gene expression data

The closest PsyB728a-homologs of the 43 putative small secreted, nonannotated proteins of PtoDC3000 proteins were identified in the PsyB728a genome. The respective expression levels of these genes during colonization of PsyB728a in the apoplast were extracted from the microarray data (dataset GSE42544 from the GEO database at NCBI), published as supplemental data of [18]. Also the expression levels of 25 type-III (T3) effectors of PsyB728a were extracted.

### Plant material and growth conditions

*Nicotiana benthamiana* and tomato (*Solanum lycopersicum* Money-Maker) were grown in a climate chamber under a 14h light (22°C) and 10h dark (18°C) cycle. *N*. *benthamiana* plants were grown until four to six week old and used for *Agrobacterium*-mediated transient protein expression (agroinfiltration).

### Detection of PtoDC3000 proteins by MS

To detect the selected PtoDC3000 proteins during infection, we inoculated *Nicotiana benthamiana* with the *ΔhopQ1-1* mutant of PtoDC3000 [48], which is fully pathogenic on this host. This pathosystem causes strong infections and sufficient apoplastic proteomes for proteomic analysis. Plants were hand-infiltrated with 10^8^ bacteria/mL and two days after inoculation the apoplastic fluids were isolated by vacuum infiltration and centrifugation. Apoplastic fluids were concentrated by methanol/chloroform precipitation, digested with trypsin and analyzed by LC-MS/MS, against the annotated PtoDC3000 proteome.

### Heterologous expression in *E*. *coli*

Cloning primers of candidate proteins (**S2 Table**) were designed in three steps. The 5’ ends of cloning fragments were defined by SignalP predictions to remove the predicted signal peptide from the candidate amino acid sequence. The 3’ ends of cloning fragments were selected from the predicted amino acid sequences to include stop-codons at the end of the sequence. From the defined positions, 20 to 22 bases of nucleotide sequences of the Pseudomonas gene were selected and fused behind Gateway adaptor sequences 5’-gggacaagtttgtacaaaaaagcaggcttgatg-3’ (forward) and 5’ggggaccactttgtacaagaaagctgggta-3’ (reverse).

To produce a Gateway-compatible pFLAG vector carrying an additional His-tag, a PCR fragment of a Gateway cassette encoding an N-terminal 6x His-tag was generated using primers 5’-atgcctcgagcaccatcaccatcaccataagcttacaagtttgtacaaaaaagctgaacg-3’ and 5’-atgctctagataccactttgtacaagaaagctgaacg-3’ using a destination vector as template, and cloned into pFLAG-ATS (Sigma-Aldrich) using XhoI and XbaI restriction enzymes, resulting in pTSGATE1 (**S1 File**).

Genomic DNA isolated from PtoDC3000 was used as PCR template. Pfu-Ultra-II polymerase (Stratagene) was used for PCR reactions according to the guidelines of the manufacturer. PCR fragments were cloned into pEntry201 vector (Invitrogen) by BP reactions according to the guidelines of the manufacturer. Cloned fragments were verified by sequencing. The LR reaction was performed to transfer inserts from pEntry201 into pTSGATE1. A list of names for pENTRY clones and pTSGATE clones is provided in **S1 Table**. Candidate expression vectors were transformed in *E*. *coli* for protein expression, either in Rossetta or BL21 strains. pTSGATE1 was tested for protein expression by cloning and expressing Avr2, EpiC1 and EpiC2B [21].

Protein expression was induced with 1 mM IPTG and proteins were purified on Ni-NTA affinity resin (Qiagen) according to the instructions of the manufacturer. Protein levels and purity was verified by protein gel electrophoresis followed by coomassie staining or western blotting using anti-FLAG antibody (Sigma) and an HRP-conjugated secondary anti-mouse antibody (Pierce). Signals were generated by chemiluminescence using the ECL Super Signal West (Thermo) and visualized on X-ray films (Kodak).

Purified proteins were dialysed with protein storage buffer (50 mM NaCl, 10 mM NaH_2_PO_4_ (pH 8) and 20% glycerol) and further concentrated using Vivaspin spin columns (3 kDa MW cut-off, Sartorius). Protein quantity was measured using RCDC protein assay (Bio-Rad). Proteins were stored in aliquots at -80°C until used for the inhibition screen.

Cip1 was recloned in pFLAG-ATS using primers 5- atgcaagcttcatcaccatcaccatcacgactacgacattcctactacggaaaacctatacttccagggccaaacgcccaagaacatcgtttcg-3’ and 5’-gcatgaattctcagttcaccgtgattgcgcactcgaaggtctg-3’ using HindIII and EcoRI restriction sites, resulting in pSK8 encoding WT Cip1 with a N-terminal, TEV-cleavable FLAG-His purification tag. Mutant Cip1 protein were subsequently generated by site-directed mutagenesis of pSK8 using primers 5’-agcaacccgggctttcgctggctgacccag-3’ and 5’-cgaaagcccgggttgctgggcagcgtgagg-3’ for the deletion mutant (ΔT) and 5’-acccggctgccggctttcgctggctgaccc-3’ and 5’-aagccgggcagccgggttgctgggcagcgtg-3’ for the substitution mutant (AA). (Mutant) Cip1 proteins were produced and purified as described above.

### Western analysis of Cip1

An antibody was raised against the Cip1 protein in rabbit by Eurogentec. The secondary anti-rabbit antibody was from sheep and conjugated to horse radish peroxidise (HRP). An overnight-grown culture of PtoDC3000 grown in Minimal Medium was centrifuged and the supernatant was used for western analysis.

### Transient expression of immune proteases

*Agrobacterium*-mediated transient expression of C14 (pTP41), Rcr3 (pTP36) and Pip1 (pTP43), was carried out as described previously [24]. Agrobacterium was grown overnight in Luria-Bertani (LB) medium containing 25 μg/mL Rifampicin and 10 μg/mL Kanamycin. The bacterial cultures were centrifuged at 4000g for 10 min and the obtained bacterial pellet was re-suspended in 10 mM MgCl_2_, 10 mM MES (pH 5) and 1 mM acetosyringone and diluted to OD_600_ = 2. The same procedure was applied to *Agrobacterium* carrying the p19 silencing inhibitor on a binary vector [49]. *Agrobacterium* cultures carrying protease genes were mixed with cultures carrying *p19* to a 1:1 ratio. After 1-2h incubation in the dark, the cultures were injected into leaves of four-to-six-week-old *N*. *benthamiana* plants using a needleless syringe. The plant material was harvested at three days after injection. In case of C14 expressing leaves, leaves were frozen in liquid nitrogen, ground to frozen leaf powder and stored at -80°C. Protein was extracted from frozen leaf material in 1 mM DTT and used for ABPP assays. In case of Rcr3 and Pip1, apoplastic fluid was isolated as described previously [24]. Ice cold water was vacuum-infiltrated into the detached leaves transiently expressing Rcr3 or Pip1. Surface-dried leaves were placed into apoplastic fluid collection tubes and centrifuged for 10 min at 2000g. Collected apoplastic fluids were transferred to microtubes, flash-frozen in liquid nitrogen and stored at -80°C. Protein concentrations were measured using the RCDC protein assay (Bio-Rad). The level of active protease was evaluated by ABPP on a dilution series.

### Competitive ABPP screen

ABPP of cysteine proteases was performed as described in [30] with small modifications. A single 30 μL standard reaction contained 100 mM NaAc (pH 6.2), 2 mM DTT, 1 μM MV201 and total extracts or apoplastic fluids from agroinfiltrated plants over expressing various proteases. For each single inhibition assay, approximately 1 μg protein purified from *E*. *coli* was incubated with the plant proteome for 30 min before adding MV201. Controls contained 50 μM E-64 (positive control) or the same volume of protein storage buffer (negative control). After adding the probe, the reaction mixture was incubated in the dark for 1h at room temperature. The reaction was terminated by adding SDS-containing sample buffer and either immediately heated at 95°C or stored at -20°C until heat denaturation. The reaction mixtures were loaded onto large 50-well, 12% SDS polyacrylamide gels and separated by electrophoresis. Fluorescent signals were detected using the Typhoon FLA9000 scanner. Signal intensities were quantified using ImageJ (http://imagej.nih.gov).

### Alignment of Cip1 with Chagasins

Genes with the following accession codes were aligned: PtoDC3000, gi28871353 (*Pseudomonas syringae* pv. *tomato* DC3000); Pph1448A, YP_276078.1 (*Pseudomonas syringae* pv. *phaseolicola* 1448A); Pta11528, gi331010052 (*Pseudomonas syringae* pv. *tabaci* 11528); PsyB728a, gi66047172 (*Pseudomonas syringae* pv. *syringae* B728a); PpW619, gi170720265 (*Pseudomonas putida* W619); Pf0-1, gi77460801 (*Pseudomonas fluorescens* Pf0-1); PmNK-01, gi330502174 (*Pseudomonas mendocina* NK-01); PaPAO1, gi553899616 (*Pseudomonas aeruginosa* PAO1-VE13); LmICP, gi28625248 (*Leishmania mexicana*); Chagasin, gi14250894 (*Trypanosoma cruzi*); EhICP2, gi122082030 (*Entamoeba histolytica*); and EhICP1, gi68056711 (*Entamoeba histolytica*).

### RT-PCR

Apoplastic fluids were isolated at 2 days upon infiltration of leaves of 4-week-old *N*. *benthamiana* with PtoDC3000(WT/Δcip1) at OD = 0.0002. The RNA was isolated from the bacterial pellet and bacteria grown in minimal medium containing mannitol and glutamate using the RNeasy mini kit (QIAGEN) according to the manufacturers protocol with an in solution DNase digest (QIAGEN) and cDNA was generated with random hexamer primers (Invitrogen) in the absence or presence of SuperscriptII reverse transcriptase (RT) following the manufacturers protocol (Thermo Fisher). PCR was performed with the primers below using Phusion polymerase (NEB) according to the manufacturer’s protocol using the program 3’ 98°C; 32 cycles of 10 sec 98°C; 20 sec 66°C; 10 sec 72°C; then 5’ 72°C. PCR products were separated on a 1.5% agarose gel, stained with ethidium bromide and detected under UV. The used primers are for PSPTO4033(recA): 5’-cggcaagggtatctacctca-3’ and 5’-ctttgcagatttccgggta-3’; PSPTO4210(Lon): 5’-gcctggacctctccaaagtc-3’ and 5’-cacttccatccggtccaaca-3’; PSPTO4211(cip1): 5’-atgccccctgttcgttttct-3’ and 5’-gaccatctccttgctctcgg-3’; and PSPTO4212(methyltransferase): 5’-agcgatctggaaattgccca-3’ and 5’-cgttggcggtgttcttcaag-3’.

### Generation of *cip1* mutants and complementation

Two different types of PtoDC3000 *Δcip1* mutants were made. UNL231 (*Δcip1*a) contains a polar mutation in *cip1*. It was made by PCR amplifying 2.0 kb upstream and downstream of *cip1* using primer set 5’-agtcggtacccgtgcgcatccgcacctggctc-3’ (which contains a KpnI site) and 5’-agtcctcgagggcaagttccggttttgcgagacg-3’ (which contains a XhoI site) and primer set 5’-agtcggatccctgtttgcgcgcggcttgtccg-3’ (which contains a BamHI site) and 5’- agtctctagagaggtgtcgctgttcatcgatgc-3’ (which contains a XbaI site), respectively. These PCR products were cloned using the indicated restriction enzyme sites in the same orientation on either side of a Sp^r^/Sm^r^ omega fragment from pHP45Ω [50] resulting in pLN3217. The insert from this construct was cloned into the suicide vector pRK415 [51] using KpnI and XbaI restriction enzymes resulting in pLN3272. pLN3272 was transformed into PtoDC3000 by electroporation. DC3000(pLN3272) transformants were grown in liquid KB medium containing spectinomycin for five consecutive days before the culture was plated onto KB plates containing spectinomycin. Homologous recombination was selected for by screening for the spectinomycin marker linked to the mutation and loss of the tetracycline marker carried on pRK415. The PtoDC3000 *cip1* polar mutant (UNL231) was confirmed by PCR to show that *cip1* gene was replaced by the omega fragment.

The second *cip1* mutant UNL232 (*Δcip1b*), which contains a non-polar mutation in *cip1* was generated using a similar strategy. The only difference was that the 2.0 kb fragments upstream and downstream of *cip1* were cloned on either side in the same orientation of an *nptII* gene in pCPP2988 [52] resulting in pLN3218. This *nptII* gene confers resistance to kanamycin but lacks transcriptional terminators. The insert containing the *cip1* mutation was cloned into pRK415 resulting in pLN3273 and this construct was electroporated into PtoDC3000. Homologous recombination of the non-polar *cip1* mutation was done as described above except selection was for the kanamycin marker linked to the non-polar *cip1* mutation. UNL232 was confirmed with PCR.

The wild type *cip1* gene under its native expression was reintroduced into UNL231 and UNL232 using a Tn7 transposon strategy described by [31] with some modifications. Briefly, DNA regions containing the *cip1* promoter and coding region were amplified with primer set 5’-caccgaattcctgccggattacctcaaaga-3’ and 5’-cataagctttcaagcgtaatctggaacatcgtatgggtagttcaccgtgattgcgcact-3’. The reverse primer contains nucleotides that encode a hemagglutinin (HA) tag. The resulting PCR fragment was cloned into pENTR/D-TOPO (Invitrogen, Carlsbad, CA) and then recombined into pUC18::Tn7-GATEWAY destination vector using LR Clonase according to the manufacturer’s instructions resulting in construct pLN6048. Construct pLN6048 was integrated into UNL231 and UNL232 by electroporation using rifampin (100 μg/ml) and gentamicin (1 μg/ml), the latter selected for strains that contained the Tn7 cassette. Primer set 5’-attagcttacgacgctacaccc-3’ and 5’-ttgaaaagagcctgccgagca-3’ was used to identify strains that contained Tn7::cip1-HA.

### *Pseudomonas syringae* infection assays

Inoculation and bacterial growth assays using PtoDC3000 were performed as previously described [53]. Briefly, for *cip1* complementation experiments 4-week-old tomato (*S*. *lycopersicum* cv. Moneymaker) plants were blunt syringe-inoculated with 10^5^ bacteria /mL and leaf disks were taken from surface-sterilized leaves at 4 h post inoculation (0 dpi), and at 3 days post inoculation. Three 1 cm^2^ leaf disks were combined and ground in 10 mM MgCl_2_ and a 1-fold dilution series was plated out on selection media. For other infection assays 4-week old tomato plants were spray-inoculated with 10^8^ bacteria /mL as described previously [54]. Leaf disks were taken from surface-sterilized leaves at 4 h post inoculation (0 dpi), and at various days post inoculation. This was repeated four times per genotype per time point per assay.

### ABPP on infected tissues

Leaves of *N*. *benthamiana* plants were untreated or infiltrated with water (Mock, M), or with *Agrobacterium tumefaciens* (OD = 0.5) carrying the P19 silencing inhibitor alone (P19), or mixed with Agrobacterium carrying C14 (C14). Two days later (2dpi), PtoDC3000(ΔhopQ1-1) was infiltrated at OD = 0.001, or water was used as mock control. Apoplastic fluids were isolated two days later (4dpi) and preincubated with and without 100 μM E-64 for 30 minutes and then labeled with 2 μM MV201 for 5 hours. Proteins were separated on 14% SDS-PAGE and the gel was scanned for fluorescence (532nm excitation, 580BP filter, 600PMT) and stained by Sypro Ruby.

### Bacterial growth *in vitro*

Wild-type and both *Δcip1* mutants of PtoDC3000 were inoculated at OD = 0.05 in LB medium without antibiotics at 28°C and bacterial growth was measured every 30 minutes for 12 hours at OD600.

### Papain inhibition assay

The inhibitory activity of Cip1 against papain (Sigma-Aldrich) was determined by assaying the proteolytic activity of 30 μl of 1 mg/ml papain in Tris-HCl buffer, pH 6.8 in the presence of 1 mM glutathione, using 1.5 mM Nα-Benzoyl-L-arginine 4-nitroanilide hydrochloride (BAPNA, Sigma-Aldrich) as the substrate in the presence or absence of Cip1. The kinetic parameters for substrate hydrolysis were determined by measuring the initial rate of enzymatic activity. The inhibition constant Ki was determined with the Lineweaver-Burk equation. The Ki value was also calculated from the double reciprocal equation by fitting the data into the computer software Origin 6.1. For the Lineweaver-Burk analysis, 1 μM papain was incubated with and without 3.98 nM and 7.46 nM inhibitor and assayed at increasing concentration of BAPNA (0.1–5 mM) at 37°C for 30 min. The reciprocals of substrate hydrolysis (1/V) for inhibitor concentration were plotted against the reciprocal of the substrate concentration, and the Ki was determined by fitting the resulting data. For comparison of Cip1 with mutant Cip1, 4.1 μM papain was incubated with 0.56 μM (mutant) Cip1 inhibitor or chicken cystatin and 0.2 mM BAPNA in a 100 μl volume. Substrate conversion was monitored by increased fluorescence at 410 nm over time using an Infinite M200 Tecan microtiter plate reader.

### Competitive ABPP with mutant Cip1

5 μg/ml papain (Sigma-Aldrich) or 10-fold diluted apoplastic fluids from *N*. *benthamiana* leaves overexpressing C14 (see above), were preincubated in a buffer containing 25 mM NaAc pH 5.0 and 2 mM DTT with 100μM E-64 or (mutant) Cip1 for 30 minutes, and then labeled with 1 μM MV201 for 1 hour. The labeling reaction was stopped by adding SDS loading buffer and boiling for 5 minutes.

### Accession codes

Rcr3 (Solyc02g076980), Pip1 (Solyc02g077040), C14 (Solyc12g088670), and Cip1 (PSPTO4211)

## Supporting Information

S1 Fig Occurence of small, secreted non-annotated putative proteins of PtoDC3000 in *Pseudomonas* species. BLAST scores were generated for each of the 131 small secreted, non-annotated proteins against each of the protein databases of 24 sequenced *Pseudomonas* species. BLAST scores were presented in shades of red, and black boxes represent no significant BLAST score. Blast scores are clustered over both the species and proteins. Conservation of the proteins occurs in five groups (1–5, right side). Representatives having the highest SP scores were picked from each of these classes and produced and purified (black boxes). *P*. *syringae* pv *tomato* (PtoDC3000); pv. *syringae* (PsyB728a); pv. *phaseolicola* (Pph1448A); pv. *tabaci* (Pta11528); *P*. *fulva* (Pfulva12-X); *P*. *mendocina* (PmNK-01 and PmYMP); *P*. *stutzeri* (PtA1501); *P*. *aeruginosa* (PaPA7, PaC3719, PaPACS2, PaLESB58, Pa39016, PaPA14, Pa2192 and PaPAO1); *P*. *brassicacearum* (PbbNFM421); *P*. *fluorescence* (Pf0-1, PfSBW25 and Pf-5); *P*. *entomophila* (PeL48); *P*. *putida* (PpGB-1, PpF1, PpW619 and PpKT2440).
(PDF)

S2 Fig Anti-FLAG western blot of purified small secreted non-annotated putative proteins. Small secreted non-annotated proteins were expressed from pTSGAT1 in *E*. *coli* as FLAG-His-tagged proteins and purified on Ni-NTA columns. Proteins were separated on protein gels, transferred onto a PVDF membrane and detected with anti-FLAG antibodies.
(PDF)

S3 Fig C14 produced by agroinfiltration. Silencing inhibitor p19 was transiently co-expressed with and without C14 upon agroinfiltration of *N*. *benthamiana*. Leaf extracts were generated and preincubated with and without E-64 and then labeled with 1 μM MV201. Proteins were separated on protein gels and detected by in-gel fluorescence scanning. To label only mature C14, extracts were centrifuged and the supernatant was taken for labeling. Abbreviations: i, iC14; m, mC14.
(PDF)

S4 Fig Both *Δcip1* mutants show reduced bacterial growth upon spray inoculation of tomato. Two independent *Δcip1* mutants (UNL231(a) and UNL232(b)) and the wild-type of PtoDC3000 (WT) were spray-inoculated onto tomato and bacterial growth (in colony-forming units, CFU) was measured shortly after inoculation (0dpi) and 1, 2 and 3 days-post inoculation (dpi). Error bars represent at least three biological replicates. This experiment was repeated twice having similar results. *, p>0.05.
(PDF)

S5 Fig *Δcip1* deletion mutants grow normally *in vitro*. WT and two independent *Δcip1* mutants were inoculated at OD = 0.05 in LB medium without antibiotics at 28°C and bacterial growth was measured every 30 minutes at OD_600_. Similar results were obtained in repetition experiments.
(PDF)

S6 Fig PtoDC3000 infection has no significant effect on protease activity in AF. Leaves of *N*. *benthamiana* plants were untreated (A) or infiltrated (B) at 0 days-post-infiltration (0dpi) with water (Mock, M), or with *Agrobacterium tumefaciens* (OD = 0.5) carrying the P19 silencing inhibitor alone (P19), or mixed with agrobacterium carrying C14 (C14). Two days later (2dpi), PtoDC3000(ΔhopQ1-1) was infiltrated at OD = 0.001 (Pst), or water was used as mock control (M). Apoplastic fluids were isolated two days later (4dpi) and preincubated with and without 100 μM E-64 for 30 minutes and then labeled with 2 μM MV201 for 5 hours. Proteins were separated on 14% SDS-PAGE and the gel was scanned for fluorescence (532nm excitation, 580BP filter, 600PMT) and stained by Sypro Ruby.
(PDF)

S7 Fig Inhibition of C14, Pip1 and Rcr3 occurs at both apoplastic and neutral pH. C14, Pip1 and Rcr3 were transiently overexpressed by agroinfiltration of *Nicotiana benthamiana* and proteomes were isolated and pre-incubated with and without 7.8 μM Cip1 at various pH for 30 minutes and then labeled with 1 μM MV201 for one hour. Labeled proteins were detected by in-gel fluorescent scanning.
(PDF)

S8 Fig Cip1 is a weak inhibitor of 34 different Rcr3 variants. 34 different Rcr3 variants representing six different wild tomato species were produced by agroinfiltration. Apoplastic fluids from leaves overexpressing these Rcr3 variants were preincubated for 30 minutes with 40μM E-64, 100 nM Avr2 or 1μM Cip1 and labeled for 4 hrs with 0.04 μM MV201. Samples were separated on protein gels and scanned for fluorescence. Signals were quantified, normalized to E-64 inhibition (= 100%) and shown as mean for each species, with the number of tested Rcr3 variants shown between brackets. Error bars indicate standard error of n different samples.
(PDF)

S1 File Nucleotide sequence of pTSGATE01. (TXT)

S1 Table Protein selection. This table contains the *in silico* selections of the small, secreted, non-annotated PSPTO proteins.
(XLS)

S2 Table Oligonucleotide sequences. This table contains the primer sequences used for PCR.
(XLS)

S3 Table Expression. This table contains the expression values for the PtoB728a genes encoding the 43 selected proteins and the type III effectors in the apoplast.
(XLS)

S4 Table Proteomics. This table summarizes the selected PSPTO proteins detected in the apoplast of plants infected with PtoDC3000.
(XLSX)
