# Supplementary material for: Screen of Non-annotated Small Secreted Proteins of Pseudomonas syringae Reveals a Virulence Factor That Inhibits Tomato Immune Proteases
Source: PLoS Pathog. 2016 Sep 7;12(9):e1005874. doi: 10.1371/journal.ppat.1005874 (PMC5014320; doi:10.1371/journal.ppat.1005874)
Supplement: S1 Fig — BLAST scores were generated for each of the 131 small secreted, non-annotated proteins against each of the protein databases of 24 sequenced Pseudomonas species. BLAST scores were presented in shades of red, and black boxes represent no significant BLAST score. Blast scores are clustered over both the species and proteins. Conservation of the proteins occurs in five groups (1–5, right side). Representatives having the highest SP scores were picked from each of these classes and produced and purified (black boxes). P. syringae pv tomato (PtoDC3000); pv. syringae (PsyB728a); pv. phaseolicola (Pph1448A); pv. tabaci (Pta11528); P. fulva (Pfulva12-X); P. mendocina (PmNK-01 and PmYMP); P. stutzeri (PtA1501); P. aeruginosa (PaPA7, PaC3719, PaPACS2, PaLESB58, Pa39016, PaPA14, Pa2192 and PaPAO1); P. brassicacearum (PbbNFM421); P. fluorescence (Pf0-1, PfSBW25 and Pf-5); P. entomophila (PeL48); P. putida (PpGB-1, PpF1, PpW619 and PpKT2440). (PDF) [file ppat.1005874.s001.pdf]

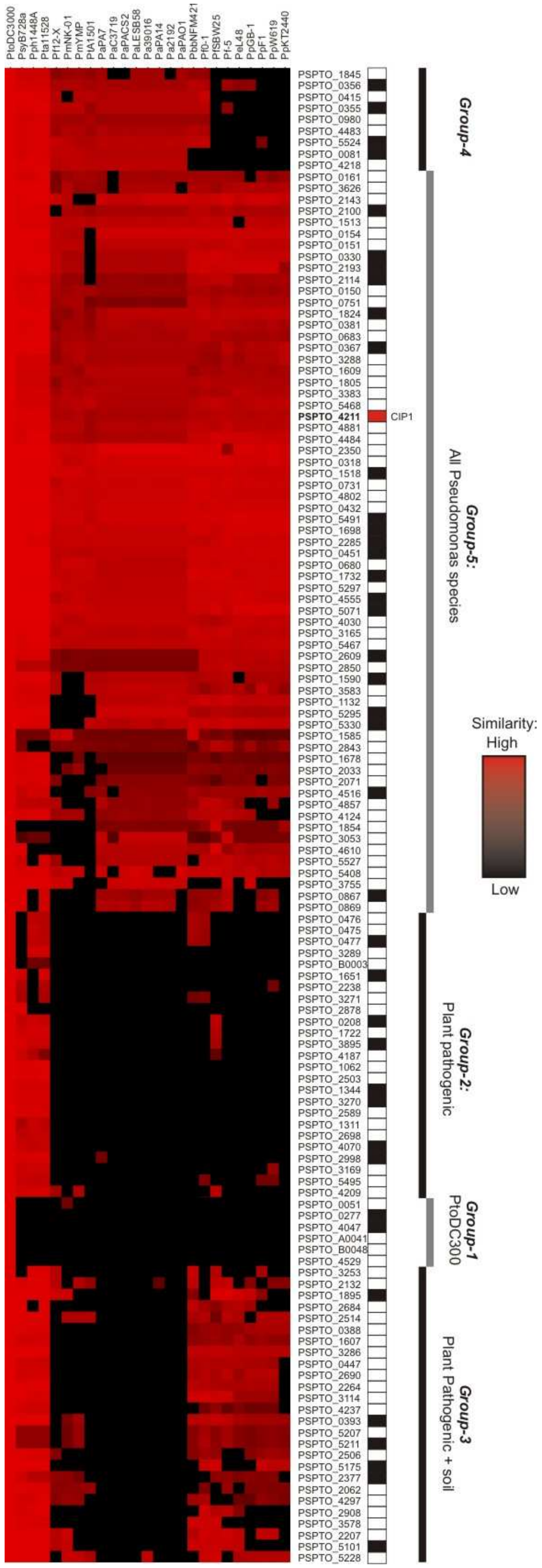

Figure S1. Occurrence of small, secreted non-annotated proteins of PtoDC3000 in *Pseudomonas* species.

BLAST scores were generated for each of the 131 small secreted, non-annotated proteins against each of the protein databases of 24 sequenced *Pseudomonas* species. BLAST scores were presented in shades of red, and black boxes represent no significant BLAST score. Blast scores are clustered over both the species and proteins. Conservation of the proteins occurs in five groups (1-5, right side). Representatives having the highest SP scores were picked from each of these classes and produced and purified (black boxes).

P. syringae pv *tomato* (PtoDC3000); pv. *syringae* (PsyB728a); pv. *phaseolicola* (Pph1448A); pv. *tabaci* (Pta11528); *P. fulva* (Pfulva12-X); *P. mendocina* (PmNK-01 and PmYMP); *P. stutzeri* (PtA1501); *P. aeruginosa* (PaPA7, PaC3719, PaPACS2, PaLESB58, Pa39016, PaPA14, Pa2192 and PaPAO1); *P. brassicacearum* (PbbNFM421); *P. fluorescence* (Pf0-1, PfSBW25 and Pf-5); *P. entomophila* (PeL48); *P. putida* (PpGB-1, PpF1, PpW619 and PpKT2440).
