# Supplementary material for: Screen of Non-annotated Small Secreted Proteins of Pseudomonas syringae Reveals a Virulence Factor That Inhibits Tomato Immune Proteases
Source: PLoS Pathog. 2016 Sep 7;12(9):e1005874. doi: 10.1371/journal.ppat.1005874 (PMC5014320; doi:10.1371/journal.ppat.1005874)
Supplement: S2 Fig — Small secreted non-annotated proteins were expressed from pTSGAT1 in E. coli as FLAG-His-tagged proteins and purified on Ni-NTA columns. Proteins were separated on protein gels, transferred onto a PVDF membrane and detected with anti-FLAG antibodies. (PDF) [file ppat.1005874.s002.pdf]

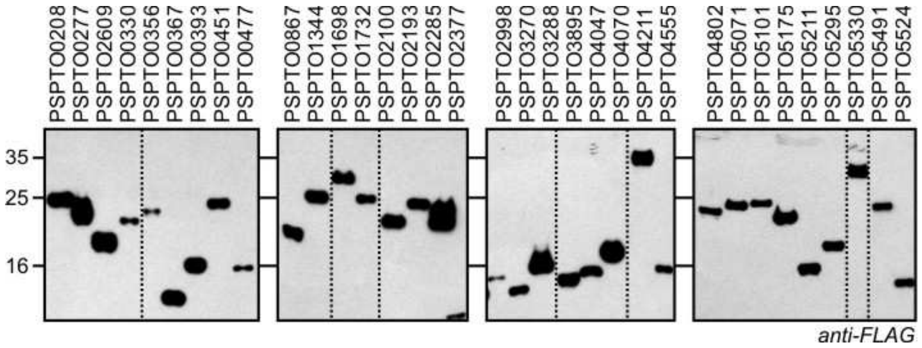

Figure S2. Anti-FLAG western blot of purified small secreted non-annotated proteins. Small secreted non-annotated proteins were expressed from pTSGAT1 in *E. coli* as FLAG-His-tagged proteins and purified on Ni-NTA columns. Proteins were separated on protein gels, transferred onto a PVDF membrane and detected with anti-FLAG antibodies.
