# Supplementary material for: Screen of Non-annotated Small Secreted Proteins of Pseudomonas syringae Reveals a Virulence Factor That Inhibits Tomato Immune Proteases
Source: PLoS Pathog. 2016 Sep 7;12(9):e1005874. doi: 10.1371/journal.ppat.1005874 (PMC5014320; doi:10.1371/journal.ppat.1005874)
Supplement: S3 Fig — Silencing inhibitor p19 was transiently co-expressed with and without C14 upon agroinfiltration of N. benthamiana. Leaf extracts were generated and preincubated with and without E-64 and then labeled with 1 μM MV201. Proteins were separated on protein gels and detected by in-gel fluorescence scanning. To label only mature C14, extracts were centrifuged and the supernatant was taken for labeling. Abbreviations: i, iC14; m, mC14. (PDF) [file ppat.1005874.s003.pdf]

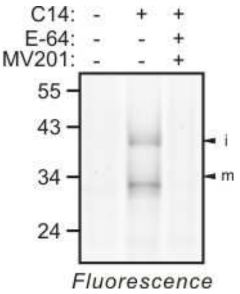

Figure S3. C14 produced by agroinfiltration.

Silencing inhibitor p19 was transiently co-expressed with and without C14 upon agroinfiltration of *N. benthamiana*. Leaf extracts were generated and preincubated with and without E-64 and then labeled with 1 µM MV201. Proteins were separated on protein gels and detected by in-gel fluorescence scanning. To label only mature C14, extracts were centrifuged and the supernatant was taken for labeling. Abbreviations: i, iC14; m, mC14.
