# Supplementary material for: Screen of Non-annotated Small Secreted Proteins of Pseudomonas syringae Reveals a Virulence Factor That Inhibits Tomato Immune Proteases
Source: PLoS Pathog. 2016 Sep 7;12(9):e1005874. doi: 10.1371/journal.ppat.1005874 (PMC5014320; doi:10.1371/journal.ppat.1005874)
Supplement: S4 Fig — Two independent Δcip1 mutants (UNL231(a) and UNL232(b)) and the wild-type of PtoDC3000 (WT) were spray-inoculated onto tomato and bacterial growth (in colony-forming units, CFU) was measured shortly after inoculation (0dpi) and 1, 2 and 3 days-post inoculation (dpi). Error bars represent at least three biological replicates. This experiment was repeated twice having similar results. *, p>0.05. (PDF) [file ppat.1005874.s004.pdf]

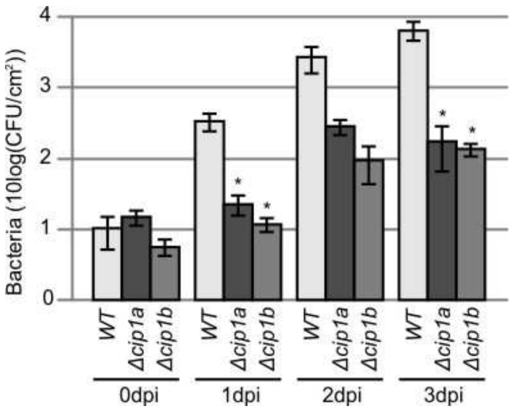

Figure S4. Both *Δcip1* mutants show reduced bacterial growth upon spray inoculation of tomato.

Two independent *Δcip1* mutants (UNL231(a) and UNL232(b)) and the wild-type of PtoDC3000 (WT) were spray-inoculated onto tomato and bacterial growth (in colony-forming units, CFU) was measured shortly after inoculation (0dpi) and 1, 2 and 3 days-post inoculation (dpi). Error bars represent at least three biological replicates. This experiment was repeated twice having similar results. *, $p>0.05$.
