# Supplementary material for: Screen of Non-annotated Small Secreted Proteins of Pseudomonas syringae Reveals a Virulence Factor That Inhibits Tomato Immune Proteases
Source: PLoS Pathog. 2016 Sep 7;12(9):e1005874. doi: 10.1371/journal.ppat.1005874 (PMC5014320; doi:10.1371/journal.ppat.1005874)
Supplement: S5 Fig — WT and two independent Δcip1 mutants were inoculated at OD = 0.05 in LB medium without antibiotics at 28°C and bacterial growth was measured every 30 minutes at OD600. Similar results were obtained in repetition experiments. (PDF) [file ppat.1005874.s005.pdf]

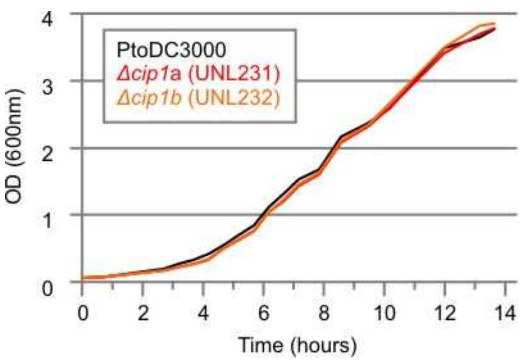

Figure S5. *Δcip1* deletion mutants grow normally *in vitro*.

WT and two independent *Δcip1* mutants were inoculated at OD=0.05 in LB medium without antibiotics at 28ºC and bacterial growth was measured every 30 minutes at OD_{600}. Similar results were obtained in repetition experiments.
