# Supplementary material for: Screen of Non-annotated Small Secreted Proteins of Pseudomonas syringae Reveals a Virulence Factor That Inhibits Tomato Immune Proteases
Source: PLoS Pathog. 2016 Sep 7;12(9):e1005874. doi: 10.1371/journal.ppat.1005874 (PMC5014320; doi:10.1371/journal.ppat.1005874)
Supplement: S6 Fig — Leaves of N. benthamiana plants were untreated (A) or infiltrated (B) at 0 days-post-infiltration (0dpi) with water (Mock, M), or with Agrobacterium tumefaciens (OD = 0.5) carrying the P19 silencing inhibitor alone (P19), or mixed with agrobacterium carrying C14 (C14). Two days later (2dpi), PtoDC3000(ΔhopQ1-1) was infiltrated at OD = 0.001 (Pst), or water was used as mock control (M). Apoplastic fluids were isolated two days later (4dpi) and preincubated with and without 100 μM E-64 for 30 minutes and then labeled with 2 μM MV201 for 5 hours. Proteins were separated on 14% SDS-PAGE and the gel was scanned for fluorescence (532nm excitation, 580BP filter, 600PMT) and stained by Sypro Ruby. (PDF) [file ppat.1005874.s006.pdf]

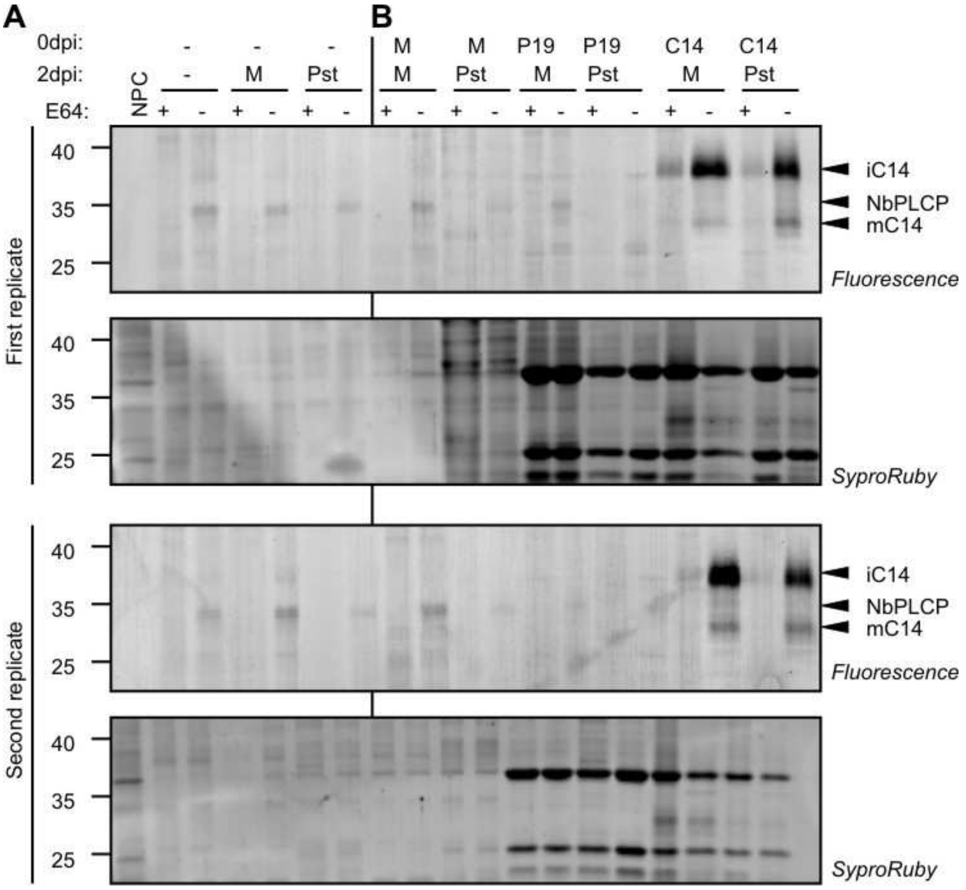

Figure S6 PtoDC3000 infection has no significant effect on protease activity in AF. Leaves of *N. benthamiana* plants were untreated (A) or infiltrated (B) at 0 days-post-infiltration (0dpi) with water (Mock, M), or with *Agrobacterium tumefaciens* (OD=0.5) carrying the P19 silencing inhibitor alone (P19), or mixed with agrobacterium carrying C14 (C14). Two days later (2dpi), PtoDC3000(ΔhopQ1-1) was infiltrated at OD=0.001 (Pst), or water was used as mock control (M). Apoplastic fluids were isolated two days later (4dpi) and preinecubated with and without 100 μM E-64 for 30 minutes and then labeled with 2 μM MV201 for 5 hours. Proteins were separated on 14% SDS-PAGE and the gel was scanned for fluorescence (532nm excitation, 580BP filter, 600PMT) and stained by Sypro Ruby.
