# Supplementary material for: Screen of Non-annotated Small Secreted Proteins of Pseudomonas syringae Reveals a Virulence Factor That Inhibits Tomato Immune Proteases
Source: PLoS Pathog. 2016 Sep 7;12(9):e1005874. doi: 10.1371/journal.ppat.1005874 (PMC5014320; doi:10.1371/journal.ppat.1005874)
Supplement: S7 Fig — C14, Pip1 and Rcr3 were transiently overexpressed by agroinfiltration of Nicotiana benthamiana and proteomes were isolated and pre-incubated with and without 7.8 μM Cip1 at various pH for 30 minutes and then labeled with 1 μM MV201 for one hour. Labeled proteins were detected by in-gel fluorescent scanning. (PDF) [file ppat.1005874.s007.pdf]

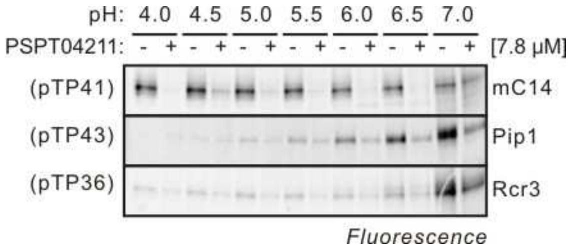

Figure S7. Inhibition of C14, Pip1 and Rcr3 occurs at both apoplastic and neutral pH. C14, Pip1 and Rcr3 were transiently overexpressed by agroinfiltration of *Nicotiana benthamiana* and proteomes were isolated and pre-incubated with and without 7.8 µM Cip1 at various pH for 30 minutes and then labeled with 1 µM MV201 for one hour. Labeled proteins were detected by in-gel fluorescent scanning.
