# Supplementary material for: Screen of Non-annotated Small Secreted Proteins of Pseudomonas syringae Reveals a Virulence Factor That Inhibits Tomato Immune Proteases
Source: PLoS Pathog. 2016 Sep 7;12(9):e1005874. doi: 10.1371/journal.ppat.1005874 (PMC5014320; doi:10.1371/journal.ppat.1005874)
Supplement: S8 Fig — 34 different Rcr3 variants representing six different wild tomato species were produced by agroinfiltration. Apoplastic fluids from leaves overexpressing these Rcr3 variants were preincubated for 30 minutes with 40μM E-64, 100 nM Avr2 or 1μM Cip1 and labeled for 4 hrs with 0.04 μM MV201. Samples were separated on protein gels and scanned for fluorescence. Signals were quantified, normalized to E-64 inhibition (= 100%) and shown as mean for each species, with the number of tested Rcr3 variants shown between brackets. Error bars indicate standard error of n different samples. (PDF) [file ppat.1005874.s008.pdf]

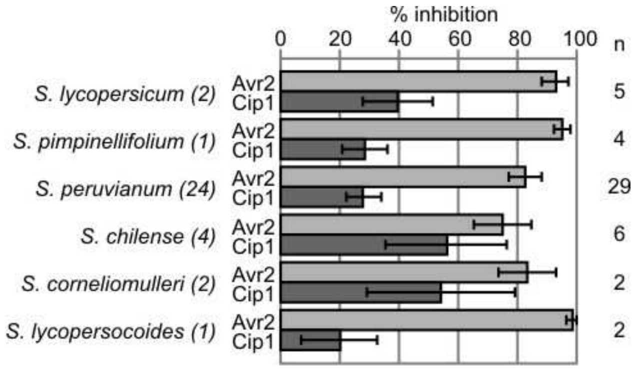

Figure S8. Cip1 is a weak inhibitor of 34 different Rcr3 variants.

34 different Rcr3 variants representing six different wild tomato species were produced by agroinfiltration. Apoplastic fluids from leaves overexpressing these Rcr3 variants were preincubated for 30 minutes with 40µM E-64, 100 nM Avr2 or 1µM Cip1 and labeled for 4 hrs with 0.04 µM MV201. Samples were separated on protein gels and scanned for fluorescence. Signals were quantified, normalized to E-64 inhibition (=100%) and shown as mean for each species, with the number of tested Rcr3 variants shown between brackets. Error bars indicate standard error of n different samples.
